# Multi-omic single-cell snapshots reveal multiple independent trajectories to drug tolerance in a melanoma cell line

**DOI:** 10.1038/s41467-020-15956-9

**Published:** 2020-05-11

**Authors:** Yapeng Su, Melissa E. Ko, Hanjun Cheng, Ronghui Zhu, Min Xue, Jessica Wang, Jihoon W. Lee, Luke Frankiw, Alexander Xu, Stephanie Wong, Lidia Robert, Kaitlyn Takata, Dan Yuan, Yue Lu, Sui Huang, Antoni Ribas, Raphael Levine, Garry P. Nolan, Wei Wei, Sylvia K. Plevritis, Guideng Li, David Baltimore, James R. Heath

**Affiliations:** 1 Division of Chemistry and Chemical Engineering, California Institute of Technology, Pasadena, California USA; 2 Division of Biology and Biological Engineering, California Institute of Technology, Pasadena, California USA; 3 Institute for Systems Biology, Seattle, Washington USA; 4 Cancer Biology Program, Stanford University School of Medicine, Stanford, California USA; 5 Department of Medicine, University of California, Los Angeles, Los Angeles, California USA; 6 Department of Molecular and Medical Pharmacology, UCLA, Los Angeles, California USA; 7 Department of Surgery, UCLA, Los Angeles, California USA; 8 Jonsson Comprehensive Cancer Center, UCLA, Los Angeles, California USA; 9 The Fritz Haber Research Center, The Hebrew University, Jerusalem, Israel; 10 Department of Microbiology and Immunology, Stanford University, Stanford, California USA; 11 Department of Radiology, Stanford University, Stanford, California USA; 12 Center of Systems Medicine, Institute of Basic Medical Sciences, Chinese Academy of Medical Sciences and Peking Union Medical College, Beijing, China; 13 Suzhou Institute of Systems Medicine, Suzhou, China; 14 Present Address: Department of Chemistry, University of California, Riverside, Riverside, California USA

**Keywords:** Lab-on-a-chip, Microfluidics, Proteomics, Tumour heterogeneity, Computational biology and bioinformatics

## Abstract

The determination of individual cell trajectories through a high-dimensional cell-state space is an outstanding challenge for understanding biological changes ranging from cellular differentiation to epigenetic responses of diseased cells upon drugging. We integrate experiments and theory to determine the trajectories that single BRAF^V600E^ mutant melanoma cancer cells take between drug-naive and drug-tolerant states. Although single-cell omics tools can yield snapshots of the cell-state landscape, the determination of individual cell trajectories through that space can be confounded by stochastic cell-state switching. We assayed for a panel of signaling, phenotypic, and metabolic regulators at points across 5 days of drug treatment to uncover a cell-state landscape with two paths connecting drug-naive and drug-tolerant states. The trajectory a given cell takes depends upon the drug-naive level of a lineage-restricted transcription factor. Each trajectory exhibits unique druggable susceptibilities, thus updating the paradigm of adaptive resistance development in an isogenic cell population.

## Abstract (web summary)

Detailed understanding of how cancer cells transition from a drug sensitive to a tolerant state is lacking. Here, using single cell proteomic and metabolic data the authors uncover that isogenic BRAF mutant melanoma cells can take two distinct paths to become tolerant to BRAF inhibition.

## Introduction

Cellular processes ranging from the development of drug-tolerant states in cancer cells to stem cell differentiation can be described as cell-state changes. Specifically, certain cancer cells that are initially responsive to targeted inhibitors that act against these oncogenic drivers^1^ can evolve into a drug-tolerant state via non-genetic mechanisms, perhaps preceding the emergence of drug-resistant clones^2–5^. The molecular details of how the cancer cells transition between the two states can inform the use of additional drugs designed to arrest the transition^6–8^. Previous studies have uncovered mechanistic insights of drug tolerance at the signaling, metabolic, transcriptional, and epigenetic levels^5,9^. However, most of these studies either compared drug-tolerant cells and drug-sensitive cells only at bulk level without single-cell resolution or did not provide a detailed time-resolved characterization of the trajectories connecting the two states. We hypothesize that there could be multiple independent paths accessible to the cells between the drug-sensitive and drug-tolerant states. If this is true, then the challenge of finding drug combinations that can arrest the unfavorable cell-state transition is significantly increased. Here we investigate a highly plastic cancer cell line that, when treated with a targeted inhibitor, switches from a rapidly dividing drug-responsive state to a drug-tolerant, slow-cycling state within a few days. We show that the cells can indeed take multiple classes of trajectories between the two states. Each trajectory class is characterized by a unique signaling and metabolic network with distinct drug susceptibilities.

From a functional perspective, cell-state changes are often accompanied by changes in gene expression^7,10–13^, protein signaling^9,10,12,14–19^, and cellular metabolism^20–23^. Highly multiplexed single-cell methods^24–27^ can provide powerful tools for mapping out cell-state landscapes associated with cell-state changes^17,28–31^. However, capturing the trajectories that individual cells take as they traverse those landscapes is challenging, even for the case of an isogenic cell line. This is because multiplex single-cell omics methods only provide snapshots of the occupied cell-state space at a given instant. Measured similarities between cells captured at successive time points can imply probable paths through the landscape^32–35^. However, cells may stochastically switch from one state to another, so an individual cell may not take a smooth trajectory between states. Time-lapse imaging methods can map individual cell trajectories, but for only two to three analytes for each cell, and so provide a limited view of the cell-state space^36–38^. Thus, the ability to extract cellular trajectories from a kinetic series of cell-state space snapshots would have a high value. Here we report on combined experimental and theoretical approaches towards addressing this fundamental challenge.

We utilize a patient-derived *BRAF*^*V600E*^ mutant melanoma cancer cell line^39^ as a model for the rapid development of drug tolerance against targeted inhibitors. Under BRAF inhibition, these highly plastic cells rapidly transit from a drug-responsive state to a drug-tolerant state^10,16^. We characterize this transition using integrated single-cell functional proteomic and metabolic assays designed to broadly sample proteins and metabolites associated with selected cancer hallmarks and cell-state-specific processes. Dimensional reduction, information-theoretic analysis, and visualization of the time-series single-cell data uncovers a complex cell-state space landscape and hints at the possibility of two distinct paths between drug-naive and drug-tolerant states. Further experiments test whether these paths constituted independent cellular trajectories. In fact, we find that even isogenic tumor cells can undertake different, independent trajectories to drug tolerance. The two trajectories are associated with distinct signaling and metabolic networks, and are independently druggable. This finding challenges the current paradigm of targeted inhibitor resistance development and also provides guidelines for assessing the value of combination therapies.

## Results

### Single-cell proteomic and metabolic analysis of BRAFi adaptation

**Fig. 1:**
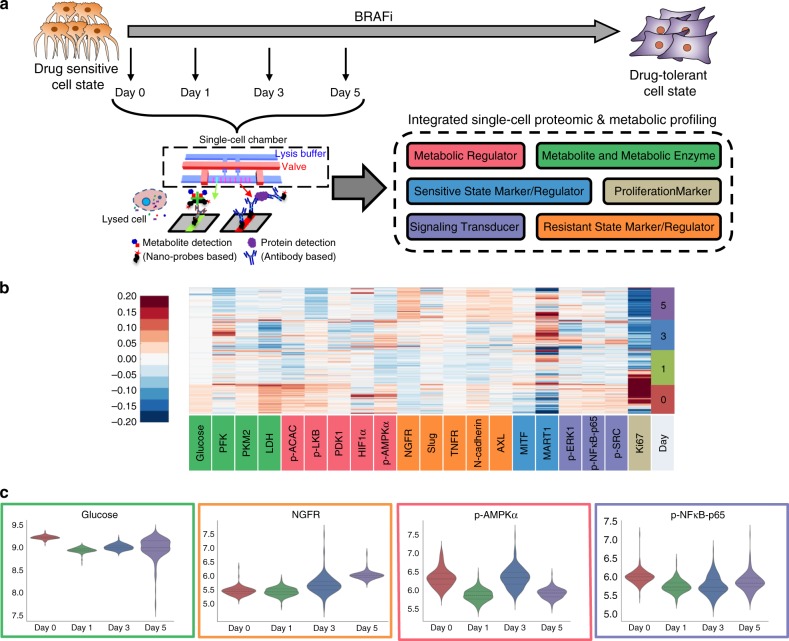
Single-cell proteomic and metabolic analysis of early drug response in M397 cells. **a** The single-cell integrated proteomic and metabolic analysis experiments design. Cells from different time points during BRAFi treatment are collected and individually analyzed using the microfluidic-based single-cell barcode (SCBC) technology. Each cell was characterized for the levels of six different categories of markers. **b** Heatmap representation of integrated proteomic and metabolic analysis dataset. Each row represents an individual cell and each column (except the last column) represents an individual analyte, with the color in the heatmap representing the measured level of the analyte. The last column represents the number of days after starting BRAFi treatment. On the X-axis, markers are colored corresponding to which of the six functional categories they belong to. **c** Violin plot representation of the distribution of certain representative markers across four time points. Y-axis represents the natural log of the measured marker level. Each plot is bordered by the color of the functional category of the measured marker.

We characterized drug adaptation in individual melanoma cells by assaying for a panel of selected proteins, plus glucose uptake, in BRAF^*V600E*^ mutant M397 cell cultures during the first 5 days of BRAFi treatment using the Single Cell Barcode Chip (SCBC)^10,17,26,40–43^ (Fig. 1a). Following 0, 1, 3, and 5 days (D0 control, D1, D3, and D5) of drug treatment, individual cells were isolated into nanoliter-volume microchambers within an SCBC. Each isolated cell was lysed in situ to release its cellular contents. Each microchamber within an SCBC contains a full barcode array in which each barcode element is either an antibody for specific protein capture^44^ or a molecular probe designed to assay for a specific metabolite via a competition assay^42,43^ (Fig. 1a). The design of this panel was informed by transcriptomic analysis of BRAFi-treated M397 cells (Supplementary Fig. 1) and existing literature^9,10,12,20,45^. The panel broadly samples various functional and metabolic hallmarks of cancer and cell-state markers.

Single-cell profiling of BRAFi-naive (D0) M397 cells revealed heterogeneous levels of many assayed markers at baseline. Referring to Fig. 1b, c and Supplementary Fig. 2, certain analytes exhibited high variability across the cell population. These include the melanocytic lineage transcription factor MITF and its downstream melanocytic cell-state marker MART1, the metabolic regulators HIF1α and p-AMPKα, and the proliferation marker Ki67. The variance in Ki67 implies that the population contains both rapid-cycling and slow-cycling cells. By contrast, high glucose uptake and the expression of metabolic enzymes lactate dehydrogenase (LDH) and PKM2 were relatively uniform from cell-to-cell. Drug treatment initially (at D1) inhibits glucose uptake and represses most metabolic regulators and signaling phosphoproteins, as well as Ki67. The repression of these cancer hallmarks reflects blockage of the key oncogenic signaling pathway upon initial BRAF inhibition. The drug also promotes transient cell differentiation followed by dedifferentiation, as evidenced by an increase of MART1 expression in D3 followed by its downregulation in D5. However, a small subpopulation of M397 cells remained Ki67-High in D1, implying a slower drug response in that subset of cells. At D3, most analytes exhibit a sharp and transitory increase in variance, which shrinks by D5. This change includes all of the metabolic regulators except p-LKB, all resistant state markers and regulators except Slug, all of the metabolic enzymes, and all of the signaling phosphoproteins. The increased magnitude of the fluctuations of many markers at D3, based upon previous reports^41,46^, implies one or more cell state changes near this time point. This was also confirmed by flow cytometry analysis (Supplementary Fig. 3). By D5, glucose uptake increased back to near D0 levels, but with increased variance. Ki67 is further decreased and with a sharply decreased variance relative to D0. In fact, most cells in D5 enter a state of senescence, without an increased incidence of apoptotic cell death (Supplementary Figs. 4 and 5). In addition, at D5, the variance and abundance of the epithelial-mesenchymal transition-related transcription factor, Slug, has increased, indicating the emergence of some cells that are trending towards a mesenchymal phenotype. Further, the levels of the other assayed protein markers that are associated with drug resistance (AXL, N-cadherin, NGFR, and TNFR) were all higher by D5. The changes of these markers were also confirmed via flow cytometry analysis (Supplementary Fig. 6). The upregulation of glucose uptake and many resistance markers indicates that cells have initiated drug resistance programs by D5. Thus, single-cell integrated proteomic and metabolic analysis, when viewed at the level of individual analytes, provides evidence of initial drug response at D1, a drug-induced cell-state change at D3, and emerging drug tolerance at D5, prior to an increase in cell proliferation (full drug resistance), which has been shown to occur a few weeks later. These observations are all consistent with the existing literature^9,12^.

### Dimensional reduction analysis implies multiple trajectories

**Fig. 2:**
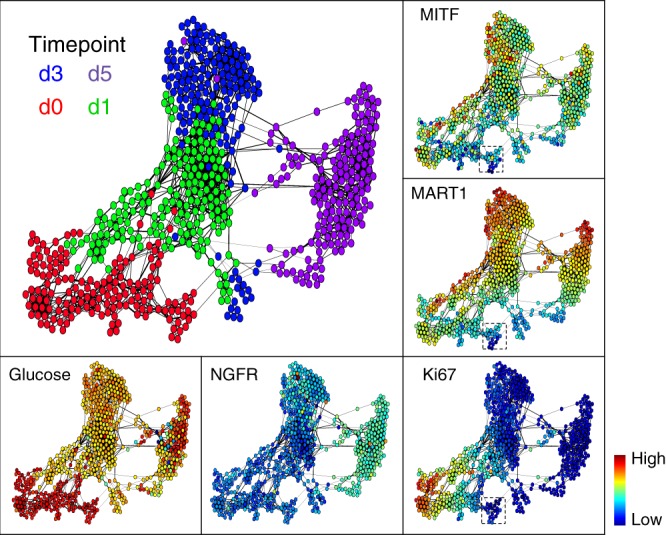
Visualization of single-cell data by FLOW-MAP. Each dot represents an individual cell. The distance between each pair of cells represents the overall multi-omic dissimilarity between them. Cell pairs that are close enough are linked with an edge in between. The colors of the dots in the main panel (upper left) represent BRAFi exposure time (0, 1, 3, or 5 days) of the corresponding cells. Dot colors in the other panels represent the abundance of each marker in each cell. The dashed-line box in the panels for MITF, MART1, and Ki67 levels show a small subpopulation of day-0 cells that are slow-cycling with less melanocytic phenotype.

Simultaneous visualization of the time-dependent, coordinated changes across multiple markers requires algorithms that can reduce the high-dimensionality of the dataset. We applied three such algorithms: the FLOW-MAP^47^, t-SNE^48^, and PHATE^49^. All approaches provided an intuitive representation of the dataset (Fig. 2 and Supplementary Figs. 7–9). FLOW-MAP analysis revealed that melanoma cells clustered primarily based upon drug exposure time (Fig. 2, upper left plot), indicating chronological cell-state trajectories. Most untreated M397 cells (in the lower left of the graph) were characterized by uniform levels of all measured analytes excepting N-cadherin, MITF, HIF1α, Ki67, and MART1 (see analyte-specific plots of Fig. 2 and Supplementary Fig. 7). Most of these non-uniformly expressed proteins exhibit differences that vary gradually from left-to-right across the D0 cluster of cells, with a small subpopulation of untreated cells (right side of D0 cluster) exhibiting lower expression of Ki67, MITF, and MART1. These features point to a small group of dedifferentiated, slow-cycling cells. Upon BRAFi treatment, the cell populations initially split to occupy two regions of the FLOW-MAP. At D1 (green points), the majority of the cells cluster to the upper right of the D0 cells, whereas a small subpopulation clusters directly to the right of the D0 group. This trend continues at D3, with most cells clustering above the largest D1 mass, while a small number cluster to the right of the small D1 group. By D5 (purple), all cells cluster to the right-hand side of the graph. The bifurcation of cells at Day 1 and 3 implies the possibility of upper and lower trajectories towards the drug-tolerant state. The possibility of two classes of trajectories was also indicated by t-SNE^48^ and PHATE^49^ analyses (Supplementary Figs. 8, 9). Thus, computational analyses of the single-cell dataset indicate a bifurcated drug response during the early stages of BRAFi adaptation.

### Surprisal analysis uncovers analyte modules of the trajectories

To further dissect the dynamics of molecular changes associated with the bifurcated drug-response trajectories, we applied surprisal analysis^50–52^ to our single-cell dataset. Surprisal analysis is a thermodynamics-inspired method that has been broadly applied to understanding large-scale bulk and single-cell omics data sets^46,50,52–54^. This approach is based on the identification of the steady state of the system (formally speaking the state of minimum free energy) and any constraints (analyte modules) that increase the free energy from this theoretical minimum^52,55^. Using this approach, we identified two main modules, each representing a set of analytes that exhibit coordinated changes across cells. The predicted expression of all 20 analytes based on these two modules matched well with the measured single-cell dataset (Supplementary Figs. 10 and 11), demonstrating that modules 1 and 2 recapitulate the overall changes of all molecular signatures across all cells over the 5-day course of drug treatment.

**Fig. 3:**
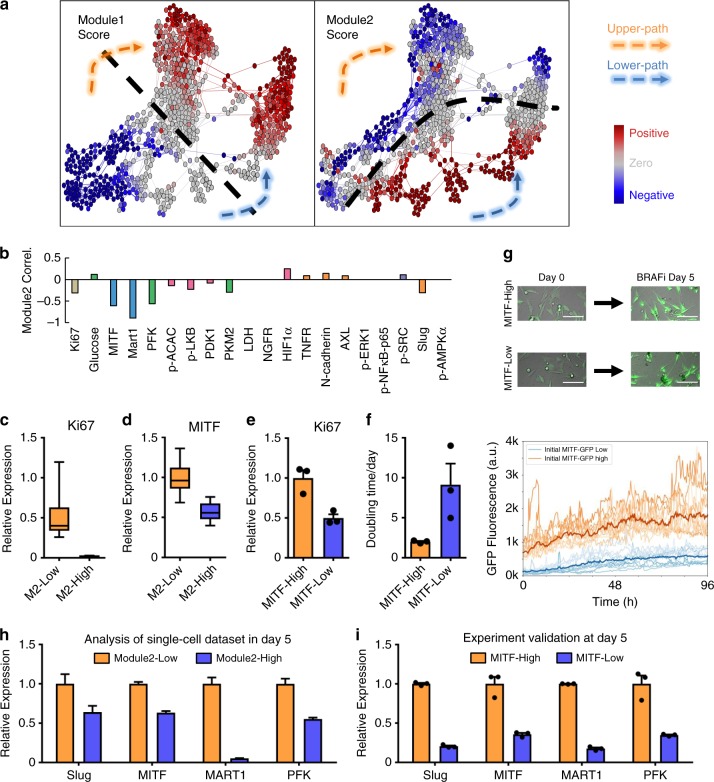
Surprisal analysis identifies MITF as a transcription factor regulating the bifurcation. **a** Visualization of the influence score of the two regulatory modules identified from surprisal analysis. Module 1 is time-dependent, whereas module 2 exhibits a path-specific pattern. The dashed black lines indicate the region for which the respective module scores of each cell approach zero. **b** Pearson’s correlation between individual marker levels and the module 2 score. **c**, **d** Boxplot of Ki67 and MITF expression level in module 2 score-high and -low subpopulations at day 0. Data are median with first and third quartiles (box), and top and bottom quartiles (whiskers) indicated. Each experiment is the result of *n* = 16 biologically independent cells per group. **e** Ki67 relative expression, measured by qPCR in sorted MITF-High and MITF-Low cells at day 0. Each experiment is the result of *n* = 3 biologically independent samples per group. **f** Doubling time measured in treatment-naive condition, collected from sorted MITF-High and MITF-Low cells at day 0. Each experiment is the result of *n* = 3 biologically independent samples per group. **g** Single-cell time-lapsed microscopy analysis of MITF-activity during 5 days of BRAFi. Top panel: time-lapse images of sorted GFP-High and GFP-Low cells before and after 5 days of BRAFi. Representative images from three biological replicates are shown. Scale bar, 100 µm. Bottom panel: single-cell MITF-reporter traces for MITF-High (orange) and MITF-Low (blue) cells. Bold lines represent the mean response. **h** Slug, MITF, MART1, and PFK relative expression levels in module 2 score-high and -low subpopulations, collected from cells at day 5 and analyzed from the single-cell dataset. Each experiment is the result of *n* = 16 biologically independent samples per group. **i** Slug, MITF, Mart1, and PFK expression, measured by qPCR in sorted MITF-High and MITF-Low day-0 cells that have been treated with BRAFi for 5 days. Each experiment is the result of *n* = 3 biologically independent samples per group. Data are presented as mean values ± SEM. Source data are provided as a Source Data file.

The influence score (the lambda values defined in ref. ^52^) of a module in a cell represents the extent to which the module-associated analytes are enriched or repressed in that cell. Modules 1 and 2 were visualized by color-coding their influence scores onto each node in the FLOW-MAP graph (Fig. 3a). We found that the influence score of module 1 gradually increased from a negative (blue) to positive (red) value along both the upper and lower paths, with a clear sign change (lambda1 = 0) in the middle time points (Fig. 3a, left panel), indicating the existence of a biophysical barrier along the transition trajectories. We have previously shown that such a sign change can imply a cell-state transition and a boundary between different cell states^50^. Considering the negative correlation of Ki67 expression and positive correlation of NGFR/AXL expression with the module 1 score (Supplementary Fig. 12), the time dependence of module 1 score change appears to reflect the transition from a drug-responsive state to a slow-cycling, drug-tolerant state between days 1 and 3. Similarly, the module 2 score, when projected on the FLOW-MAP, also exhibits a sign change (lambda2 = 0), which indicates the existence of one biophysical barrier separating the upper and lower paths (Fig. 3a, right panel). Notably, the expression of melanocytic phenotype transcription factor MITF and its downstream protein MART1 both showed negative correlations with module 2 score (Fig. 3b and Supplementary Fig. 13), indicating that the separation of the two paths may be related to the melanocytic lineage of the cells. Similar results were achieved by either additional *z*-score normalization or deleting the top two most variable markers, Ki67 and MART1 (Supplementary Fig. 14). In summary, surprisal analysis resolves both time-dependent and path-specific modules. It also reveals that, as the cells advance from drug-naive to drug-tolerant, they occupy a rather complex landscape: comprising four distinct cellular states separated by two biophysical barriers (Supplementary Fig. 15).

### Experimental validation of the bifurcated trajectories

Surprisal analysis provides theoretical support for the existence of both the upper and lower paths from drug-naive to drug-tolerant cell states. However, experimental validation is required to determine whether individual cells would follow a single trajectory along one path or the other, or if cells stochastically switch between paths. The map of module 2 on the D0 cell data hints at biological differences that separate even the untreated D0 cells into two subpopulations (State1 and State2) (Supplementary Fig. 15). The expression levels of the transcription factor MITF and its direct downstream target MART1 are among the top four markers that distinguish the two D0 subpopulations (Supplementary Fig. 16). This finding suggests that drug-treated MITF-Low cells might follow the lower path, while MITF-High cells might follow the upper path (Supplementary Figs. 17a). We thus generated MITF-green fluorescent protein (GFP) reporter cell lines and sorted GFP-High (MITF-High) and GFP-Low (MITF-Low) subpopulations (Supplementary Figs. 17b and 18). Consistent with our hypothesis, MITF-High cells displayed higher level of Ki67 and MITF, as well as a shorter doubling time relative to sorted MITF-Low subpopulations (Fig. 3c–f). This data is consistent with reported observations of melanoma phenotype switching from a melanocytic, highly proliferative state to a non-melanocytic, more invasive state^56^. It also confirmed that the two subpopulations in D0 cells can be separated using this reporter system. We next performed time-course experiments on the MITF-High and MITF-Low subpopulations to analyze the expression of the following markers individually within the two subpopulations, including Ki67, MART1, p-ERK, NGFR, AXL, and MITF by flow cytometry. Visualization of the trajectory of both subpopulations from the high-dimensional space onto three-dimensional (3D) or two-dimensional (2D) space showed a clear separation of the two trajectories (Supplementary Fig. 19). These data indicate that, even in an isogenic cell line, different subpopulations could behave differently upon BRAFi treatment. MITF-High and MITF-Low subpopulations may represent cells destined to follow the upper and lower paths, respectively, following drug treatment.

To quantify the frequency of stochastic interconversion between the sorted MITF-High and MITF-Low subpopulations during the drug treatment, we monitored the MITF activity within large numbers of single cells, over a 5-day period of BRAFi treatment. As expected, the MITF-High cells displayed higher activity (quantified by the GFP reporter) than did the MITF-Low cells (Fig. 3g), with no significant stochastic switching between the two trajectories observed.

To further confirm that the sorted cells reach their respective destination states after 5 days of drugging, we quantified the markers that are differentially expressed between the upper and lower paths at D5. Mining of the single-cell data sets revealed that several markers, including Slug, MITF, MART1, and PFK, are differentially expressed between the two paths (negative- and positive-valued module 2) at D5 (Fig. 3h and Supplementary Figs. 13 and 20a). By analyzing the expression of these four genes in sorted MITF-High and MITF-Low D0 cells after 5 days of treatment (Supplementary Fig. 20b), we found that their expression levels in sorted MITF-Low cells were significantly lower than those in MITF-High cells after 5 days of treatment (Fig. 3i). These results experimentally support that, upon drug treatment, MITF-High and MITF-Low cells take distinct trajectories towards drug tolerance along the upper and lower paths, respectively (Supplementary Fig. 20a, left panel).

### MITF is a molecular driver for the bifurcated trajectories

MITF is suggested to be an elicitor of intrinsic drug tolerance^57^. To investigate whether MITF drives the bifurcation in drug response, we generated an M397 cell line with MITF stably knocked down. Before treatment, knockdown of MITF induced the cells to become slow-cycling with characteristic low levels of *Ki67* (Supplementary Fig. 21a, b), suggesting that the downregulation of MITF will force these cells to transition along the lower path. Furthermore, upon 5 days of BRAFi treatment, MITF-knockdown cells showed significantly lower levels of *SLUG*, *MITF*, *MART1*, and *PFK* relative to control (Supplementary Fig. 21c), suggesting that MITF-silenced cells did, in fact, follow a trajectory along the lower path. Thus, MITF is identified as an important molecular driver that discriminates between the two drug-response trajectories we identified.

### Critical point analysis identifies central trajectory regulators

The tipping point is the critical point in an evolving situation that leads to a new and irreversible development^58^. Critical point analysis has been widely used in understanding state transitions in physical systems. Recently, more studies have applied critical point analysis for investigations of cell-state transitions in biological systems^46,59,60^. During a cell-state transition, there will be the tipping point at which critical changes of cell state take place. If two cell states are separated by a barrier, then the tipping point can be understood as the peak of the barrier, beyond which the cell will irreversibly transition towards a new state (Supplementary Fig. 22a). Identification of such tipping points is essential to mine the important regulators, which can drive the transition. Drugging these regulators may provide a strategy for stopping such transition (Supplementary Fig. 22b)^10,28,46,53,59,61^.

**Fig. 4:**
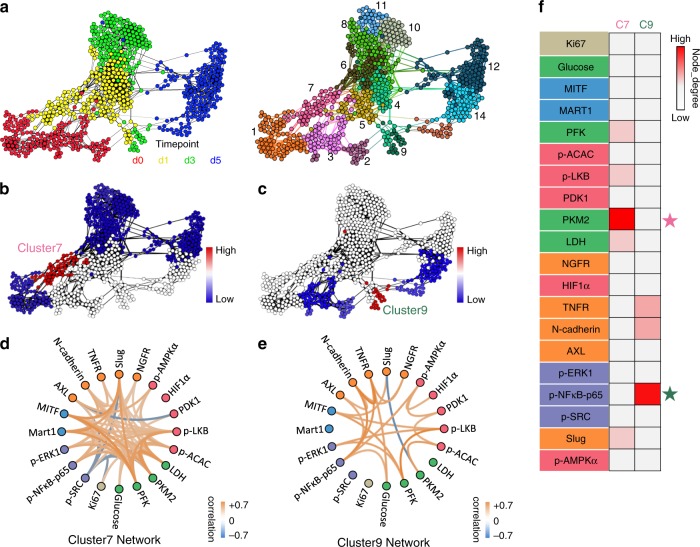
Critical point analysis and network analysis of two trajectories. **a** Clustering of all cells into four time point-defined subpopulations. The left panel is FLOW-MAP with cells color-coded by drug exposure time. The right panel is FLOW-MAP with cell color-coded as one of the 14 subpopulations defined from clustering analysis. **b** Critical point transition analysis for the upper path. Critical point index SNAI is calculated within each subpopulation associated with the upper path and color-coded onto the FLOW-MAP. Red indicates a higher SNAI value, while blue represents a lower SNAI value. Cluster 7, shown where labeled, shows the highest SNAI value in the upper path. **c** Critical point transition analysis for the lower path. Critical point index SNAI is calculated within each subpopulation associated with the lower path and color-coded onto the FLOW-MAP. Red indicates higher SNAI value, whereas blue represents lower SNAI value. Cluster 9, shown where labeled, shows the highest SNAI value in the lower path. **d** Marker–marker correlation networks, extracted from SCBC data within cluster 7 cells. The correlation strengths are reflected in the color of each edge (orange indicates positive correlation and blue indicates negative correlation). **e** Marker–marker correlation networks, extracted from SCBC data within cluster 9 cells. The correlation strengths are reflected in the color of each edge (orange indicates positive correlation and blue indicates negative correlation). **f** Importance score of each node within each network, as defined by node degree (a quantification of connectivity of a node within a network). Colors indicate the node-degree value of each node within cluster 7 or cluster 9 networks. Nodes with high scores were hypothesized to be important and some of them labeled with stars were further tested with drug perturbation.

Surprisal analysis of our single-cell data sets indicates that both the upper and lower paths are characterized by a cell-state transition (sign change of module 1 score) in the D1–D3 time window (Fig. 3a, left panel). To identify the tipping points along each of the two paths, we first clustered the single-cell data from all time points into 14 different sub-clusters on the FLOW-MAP (Fig. 4a). This overall analysis assumes that each cluster on the FLOW-MAP represents an intermediate state along the transition. Clusters 1, 6, 7, 8, 10, 11, and 12 align with the upper path, whereas clusters 2, 3, 9, 13, and 14 fall along the lower path (Fig. 4a). As there are two paths connecting day 0 and day 5 cells, we would expect there to be two tipping points, one along each path. Therefore, each cluster is assumed to contain cells at locations that are of varying distances from the critical point along its own path. It has been well-documented that a tipping point along a critical-point transition, when analyzed with single-cell resolution, exhibits a decrease of correlation between cells and a concomitant increase of correlation between genes^60^. This feature allows using a quantitative index for predicting critical transitions in a high-dimensional state space. The signaling network activity index (SNAI)^10^ and the critical transition index (Ic)^60^, which are both formalized based on such quantitative features, are two published indices used to identify regions near tipping points. Using these indices, we found cluster 7 in the upper path and cluster 9 in the lower path showed the highest values of these indices within their respective path (Fig. 4b, c, and Supplementary Figs. 23–25), suggesting that clusters 7 and 9 are closest to the tipping points along each of the two paths.

To mine the key regulators driving the cell-state transitions, we next performed network analysis^10,17,40^ for the tipping points in clusters 7 and 9. Our hypothesis is that regulators displaying higher connectivity within the network should be the crucial hub regulators that maintain the network. As a result, drugging those hub regulators can more effectively disrupt the network and therefore prevent the transition through the critical point to the drug-resistant state (Supplementary Fig. 22b). These two networks (for cluster 7 and cluster 9) are characterized by different structures (Fig. 4d, e), implying that these transitions are regulated in different ways. To identify the hub regulators of the network, which can be potential drug targets, we quantified the connectivity of each analyte (node) in the correlation networks by calculating the node degree and hub score for each node (see Methods). For cluster 7 (upper path), we found that several transcription factors and metabolic enzymes, including MITF, PFK, p-LKB, PKM2, LDH2, and Slug, showed high levels of network participation (connectivity) by both scoring metrics (Fig. 4f and Supplementary Fig. 26). For cluster 9 (lower path), TNFR, N-cadherin, and p-NFκB-p65 appeared dominant (Fig. 4f and Supplementary Fig. 26). An interesting observation was that the markers that exhibited a high score in cluster 7 often displayed a low score in cluster 9 and vice versa, indicating that the two paths are dissimilarly regulated. Importantly, these critical point analysis results are relatively stable across a range of clustering numbers (Supplementary Fig. 27).

**Fig. 5:**
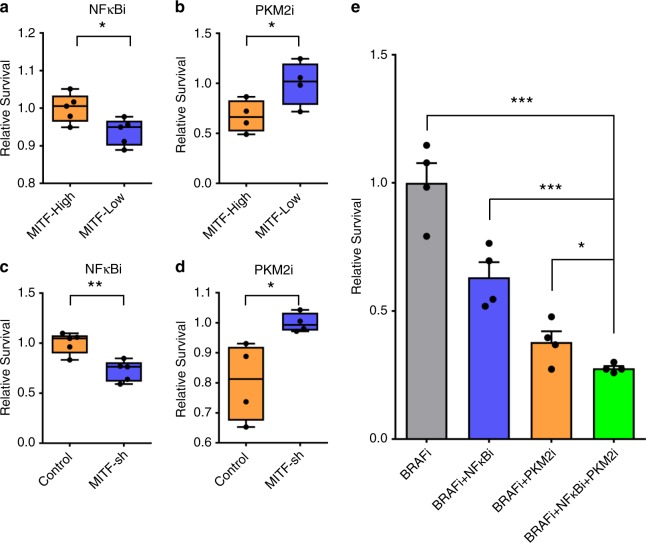
Differential drug sensitivity of cells associated with two trajectories. **a** MITF-GFP reporter cell line was sorted for MITF-High and MITF-Low subpopulations before drugging. The sorted cells were then treated with BRAFi + NFκBi combination for 5 days and then collected for cell number counting. Relative cell survival of sorted MITF-High and MITF-Low cells after undergoing BRAFi + NFκBi combination therapy for 5 days were plotted. Survival data were normalized to the MITF-High sample. Each experiment is the result of *n* = 4 biologically independent samples per group. **b** MITF-GFP reporter cell line was sorted for MITF-High and MITF-Low subpopulations before drugging. The sorted cells were then treated with BRAFi + PKM2i combination for 5 days and then collected for cell number counting. Relative cell survival of sorted MITF-High and MITF-Low cells after undergoing BRAFi + PKM2i combination therapy for 5 days were plotted. Survival data were normalized to the MITF-Low sample. Each experiment is the result of *n* = 4 biologically independent samples per group. **c** MITF-knockdown cells and control cells were treated with BRAFi + NFκBi combination for 5 days and then collected for cell number counting. Relative cell survival of sorted control and MITF-sh cells after undergoing BRAFi + NFΚBi combination therapy for 5 days were plotted. Survival data were normalized to the control sample. Each experiment is the result of *n* = 5 biologically independent samples per group. **d** MITF-knockdown cells and control cells were treated with BRAFi + PKM2i combination for 5 days and then collected for cell number counting. Relative cell survival of sorted control and MITF-sh cells after undergoing BRAFi + PKM2i combination therapy for 5 days were plotted. Survival data were normalized to the MITF-sh sample. Each experiment is the result of *n* = 4 biologically independent samples per group. For boxplots in **a**–**d**, data are median with first and third quartiles (box), and top and bottom quartiles (whiskers) indicated. **e** M397 cell treated with BRAFi, BRAFi + NFΚBi, BRAFi + PKM2i, and BRAFi + NFκBi + PKM2i for 5 days were collected for cell number counting. Relative cell survival of cells after undergoing BRAFi, BRAFi + NFκBi, BRAFi + PKM2i, or BRAFi + PKM2i + NFκBi therapy for 5 days were plotted. Survival data were normalized to cells undergoing BRAFi monotherapy treatment. Each experiment is the result of *n* = 4 biologically independent samples per group. The *P*-value was determined by a two-tailed unpaired Student’s *t*-test, *P < 0.05, **P < 0.01, ***P < 0.001. Data are presented as mean values ± SEM. Source data are provided as a Source Data file.

To examine whether the transitions along the two paths are driven by distinct hub regulators, we chose to use two commercially available compounds that specifically target PKM2 or nuclear factor-κB (NFκB) to perturb the respective hub nodes identified within clusters 7 and 9. We hypothesized that inhibition of the glycolysis enzyme PKM2 and the signaling phosphoprotein p-NFκB-p65 would differentially influence the transitions along the upper and lower paths respectively (Fig. 4f and Supplementary Fig. 26). Accordingly, we used a PKM2 inhibitor (PKM2i) or an NFκB inhibitor (NFκBi) in combination with the BRAFi to treat sorted MITF-High and MITF-Low cell subpopulations. Consistent with our hypothesis, the MITF-Low subpopulation was more sensitive to the BRAFi + NFκBi combination (Fig. 5a), whereas the MITF-High subpopulation was more sensitive to the BRAFi + PKM2i combination (Fig. 5b). This hypothesis was further validated by testing the same drug combinations on the MITF-knockdown cell line relative to unmodified M397 cells (Fig. 5c, d). Thus, cells passing along the different trajectories displayed differential sensitivities to PKM2 and NFκB inhibition.

Considering the differential regulator dependence of the two trajectories, we further hypothesized that co-blocking both trajectories by simultaneously inhibiting PKM2 and NFκB signaling might show additive effects in preventing the transitions towards BRAFi tolerance. To test this hypothesis, we used the triple-drug combination (BRAFi + PKM2i + NFκBi) to treat the M397 cells in vitro for 5 days and compared the resulting cell number against monotherapies (BRAFi only) and double-drug combinations (BRAFi + PKM2i and BRAFi + NFκBi) for 5 days. Consistent with our prediction, the triple-drug combination significantly outperformed the double-drug combinations, which in turn were superior to the monotherapy (Fig. 5e). Further, PKM2i or NFκBi monotherapy showed minimal growth inhibition on the M397 cells (Supplementary Fig. 28), implying that these drugs likely function by selectively blocking the BRAFi-induced cell-state transitions to the drug-tolerant state. These results demonstrate that the upper and lower paths are independent, have different regulators, and are independently druggable.

## Discussion

We explored here whether cell trajectories connecting the initial and final states of a cell-state transition could be determined from a kinetic series of static snapshots of the traversed cell-state space landscape. As a model system, we utilized a highly plastic, patient-derived M397 *BRAF*^*V600E*^ mutant melanoma cell line, which has been shown to reversibly transition between drug-naive and drug-resistant states upon treatment with a BRAF inhibitor^8,10,62^. Although single-cell omics tools have proven immensely valuable for resolving the cellular heterogeneity of tissues at a single given time point, here we sought to quantitatively connect that cellular heterogeneity to dynamic heterogeneity of cell-state changes.

We utilized microfluidic-based SCBC technology to characterize the cellular heterogeneity during the first 5 days of drug response. As both metabolic activity and signaling pathways display functional changes during the early drug response, the SCBC is uniquely suited here, as it is capable of simultaneously capturing both metabolites and cytoplasmic proteins (and phosphoproteins) from single cells. However, unlike single-cell RNA sequencing, single-cell proteomics is typically limited to assaying only tens of functional proteins and metabolites. To accurately capture the cell-state space accessed by M397 cells under BRAFi treatment, we first utilized transcriptomic analysis and literature guidance to define a panel of 20 analytes that included phenotypic markers, and markers of metabolic activity, oncogenic signaling, and cell proliferation, all of which are altered during the initial drug response. Single-cell analysis using this curated panel readily resolved the complex cell-state space traversed by the cells during the first few days of BRAFi treatment. Of course, moving towards larger numbers of analytes would certainly provide for a deeper characterization^63–65^.

We utilized computational and theoretical methods^29,32,33,48^, integrated with additional cell biology experiments, to infer single-cell trajectories from the SCBC kinetic series of snapshots. Dimensional reduction of the dataset using the FLOW-MAP algorithm suggested that the cells might take one of two paths (labeled as upper and lower) through cell-state space that connected the drug-naive cells to the drug-tolerant cells. Surprisal analysis of that same data resolved both a time-dependent module and a path-dependent module. The path-dependent module implied that cells traveling along one path are separated from the other path by a biophysical barrier, which appeared to be associated with the transcription factor MITF and its downstream melanocytic marker MART1. These analyses further predicted that the trajectory a specific cell takes is determined by its MITF level prior to drug treatment. These predictions were verified experimentally, which supported the integration of computational visualization methods with theoretical biophysical approaches to gain insight into a complex biological system. Such an approach should be broadly applicable to other dynamic, complex biological systems, including studies of cellular differentiation, tumorigenesis, and more.

Proliferative and invasive phenotypes are well-known in melanoma^56,66^. MITF, MART1, and Ki67 have been reported as robust markers for distinguishing these two phenotypes^56,66^. We have found that these two distinct phenotypes can co-exist even in the untreated, isogenic M397 cell line used in our study. The MITF-High and MITF-Low subpopulations not only displayed different doubling time without BRAFi treatment but also followed distinct drug-response trajectories upon treatment. These findings are consistent with the observations of melanoma cancer cell phenotype switching from a melanocytic and highly proliferative state to a non-melanocytic and more invasive state^59^. In that study, proliferative or invasive cell lines displayed fixed gene expression profiles in culture, but when transplanted in vivo, each class generated heterogeneous tumors containing cells with both kinds of expression profiles. Consistent with that observation of fixed gene expression profiles in vitro, we did not observe significant interconversion between cells traveling along different paths during the 5-day treatment period. These findings suggested that these two phenotypes are relatively stable over a few days of BRAFi treatment in vitro. Of course, our in vitro study may not fully recapitulate in vivo melanoma biology in which the tumor microenvironment can wield a strong influence. Analysis of clinical samples from early BRAFi-treated melanoma patients might further validate the clinical significance of our findings. We have previously reported that upon BRAFi treatment, the cells start with transient differentiation to early drug-tolerant state (slightly increased MITF and MART1 expression at D3). Prolonged BRAF inhibition (3 weeks) yielded a stem-cell-like drug-resistant state characterized by a sharp increase in cell proliferation and loss of MITF and MART1 expression^8,10^. Consistent with these findings, we showed that D5 cells (the early drug-tolerant state) display a low level of proliferation marker Ki67 under BRAFi. Most of these cells still express MART1 and MITF, but already start initiating the resistance-associated gene program (upregulation of glucose uptake and many stem-cell markers, e.g., AXL and CDH2). Consistent with the previous results from bulk studies^67^, we found both MITF-High and MITF-Low subpopulations will become more melanocytic after short-term drug treatment. These indicate that the transient differentiation towards a more melanocytic state may be a general early-acting mechanism that melanoma cells utilize in response to BRAF inhibition, in spite of their initial cell states. Furthermore, we also found that the transition towards MITF-Low invasive-like phenotype can be easily induced by knockdown of the MITF transcription factor. This indicates that the complex cell-state landscape is likely regulated by very few master regulators. It also emphasizes the importance of MITF as a molecular driver in regulating melanoma phenotype determination. These findings add significantly to our understanding of melanoma phenotype regulation and are uniquely revealed through single-cell analytics.

Our single-cell analysis showed that untreated cells contain both MITF-Low and MITF-High cell subpopulations, which tend to take different paths to develop drug tolerance. Thus, it is likely that the initial state of a cell would determine which path this cell may undertake. The coexistence of two distinct drug-response trajectories even in an isogenic cell line may explain the so-called mixed responses, which is commonly observed during the therapeutic treatment of melanoma patients. Such alternative escape paths may also explain why melanomas are so refractory to BRAFi-targeted therapy. Intriguingly, for each of the two paths, different drug susceptibilities were identified by critical point analysis and network analysis: the upper path was found to be susceptible to inhibition of the glycolysis enzyme PKM2, whereas the lower path is sensitive to NFκb-p65 inhibition. These differential drug sensitivity results are also consistent with previous bulk studies on invasive phenotypes of melanoma: MITF-low, invasive (or mesenchymal) melanoma cells have been reported to be more dependent on NFκB signaling^10^, and the single-cell resolution of our study reveals the exact molecular and cellular dynamics behind that observation. Co-inhibition of PKM2 and NFκB pathways demonstrated superior effects in inhibiting tumor growth; however, both genes are essential regulators in normal cells and their inhibition may cause toxicity to non-malignant tissue. Of note, the expression level of MITF has been shown to correlate with BRAFi sensitivity^68^. Thus increased dosage of BRAFi from the current cytostatic level to a cytotoxic level may eliminate the MITF-High subpopulation and its respective path. In conclusion, the resolved heterogeneous drug-response trajectories update the current understanding of resistance development and can provide a powerful methodology for identifying effective therapy combinations.

## Methods

### Cell lines, reagents, and cell culture

Patient-derived melanoma cell line, M397, used in this study was previously generated under UCLA IRB approval number 11–003254^39^. Cells were cultured at 37 °C with 5% CO_2_ in RPMI 1640 with l-glutamine (Life Technologies), supplemented with 10% fetal bovine serum (Omega), and 0.2% antibiotics (MycoZapTM Plus-CL from Lonza). The cell line was periodically authenticated to its early passage using GenePrint® 10 System (Promega). The presence of mutations in the genes of interest was checked by OncoMap 3 or Iontrone, and was confirmed by PCR and Sanger sequencing. BRAF inhibitor (vemurafenib), PKM2i (Compound 3 K), and NFκBi (JSH-23), all from Selleck Chemicals LLC, were dissolved in dimethyl sulfoxide (DMSO) at designated concentrations before applying to cell culture media. M397 cells were plated in 10-cm tissue culture plates at 60% confluency and treated with 3 µM BRAF inhibitor for the specified numbers of days.

### Microchip fabrication and integrated single-cell proteomic and metabolic assay

DNA microarrays within each microchamber were converted to antibody or Nano-probe microarrays by flowing the DNA–antibody or DNA–probe conjugate cocktail solution immediately before use. We washed the dead cells with phosphate-buffered saline (PBS) before trypsinization at respective time points. Collected cells were treated with Gluc-Bio before randomly loaded into microchambers within the SCBC for analysis. Each microchamber has an assay component and a separate reservoir of lysis buffer, and was photographed after cell loading. The SCBC was then cooled on ice for cell lysis. Following a 2 h protein and metabolite capture period at room temperature, the microchambers were flushed and the captured protein or metabolite on the arrays were converted into fluorescent readout and digitized by a Genepix scanner (Molecular Devices).

### Data processing from Genepix scanner

By a custom MATLAB code, the average fluorescence signals for all bars within a given barcode were extracted and matched with the micrograph of that array to prepare a table that contains the microchamber address, the numbers of cells, and the measured fluorescence levels of each assayed protein or metabolite. The SCBC readouts from the microchambers with a single cell were collected to form an *m* × *n* matrix table where each row (*m*) represents a specific microchamber address and each column (*n*) represents the abundance of a specific analyte. This matrix table is used for further analysis. One hundred and fifty-six, 185, 162, and 171 single cells are analyzed for day 0, day 1, day 3, and day 5 respectively.

### FLOW-MAP visualizations

All FLOW-MAP visualizations were created with the FLOWMAPR R package (version 1.2.0) available on GitHub (https://github.com/zunderlab/FLOWMAP/). Graphs were produced with seed.X = 1 and no clustering or downsampling. Final figures were produced in Gephi (https://gephi.org/) either using the bluered palette described in the FLOWMAPR package or using the jet rainbow palette. The code used to generate the exact FLOW-MAP graphs is available upon request.

### Surprisal analysis

To identify the cell-state boundaries, we thought to use surprisal analysis to deconvolute the change of many markers across cells into the change of just a few modules. Each module represents a group of markers that are collectively changing together from cell to cell. Such analysis can greatly simplify the complexity of the changes and narrow it down to just a few modules which can then be further dissected for detailed biological discovery. Computationally, surprisal analysis was applied as previously described^52^. Briefly, the measured level of analyte *i* at cell *c*, ln *X*_*i*_ (*c*), is expressed as a sum of a steady-state term ln *X*_*i*_^0^ (*c*) and several constraints (modules) *λ*_*j*_ (*c*) × *G*_*ij*_ representing deviations from the steady state. Each deviation term is a product of a cell-dependent weight (influence score) of the constraint *λ*_*j*_ (*c*) and the cell-independent contribution of the analyte to that constraint (module) *G*_*ij*_. To implement surprisal analysis, we compute the singular value decomposition of the matrix ln *X*_*i*_ (*c*). This factors this matrix in a way that determines the two sets of parameters that are needed in surprisal analysis: the Lagrange multipliers (*λ*_*j*_) for all constraints (modules) at a given time point, and for all times and the *G*_*ij*_ (time-independent) analyte patterns for all analyte *i* at each constraint *j*. In Fig. 3, cells with the top 10% most positive module 2 score are defined as Module2-High cells and the most negative 10% ones are defined as Module2-Low cells.

### Time-lapse microscopy

Movies were acquired on an Olympus IX8 inverted fluorescence microscope with hardware autofocus (ZDC2) and an environmental chamber maintaining a 37°C, 5% CO_2_ culture environment. Automated acquisition software (METAMORPH, Molecular Devices) was used to acquire differential interference contrast (DIC) and GFP images every 15 min from multiple stage positions.

### Quantification of single-cell trace

Nuclear staining images were segmented using open-source software ilastik (version 1.3.2) to acquire segmented nuclear bodies. Five frames (out of 193 frames) were used as the training set for image segmentation of each position. Pixel Classification feature of ilastik 1.3.2 was used to segment pixels of all 193 frames into “Background” and “Cell.” Then segmented h5 files, together with raw nuclear staining movie, were used in ilastik “manual-tracking workflow” to obtain 110 single-cell nuclear traces from 18 movie positions. Based on the cell-tracking results, GFP fluorescence data (background subtracted) of all 107 single-cell traces were extracted from the corresponding GFP images using a custom Python code. The 107 single-cell GFP traces were then sorted in the descending order by mean GFP level of the first 50 frames. Among them, the top 12 traces as “initial MITF-GFP-high” group and the bottom 12 traces as “initial MITF-GFP-low” group were plotted in Fig. 3g.

### Single-cell clustering

Prior to clustering, all single-cell data were separated by time point (i.e., day 0, day 1, day 3, and day 5). Rclusterpp clusters then applied which cluster the cells into 14 subpopulations. The cluster number was determined using the “elbow methods” which is based on the total within sum of squares metric^69^. Rclusterpp clusters were produced using the Rclusterpp R package (version 0.2.5), using all default settings (https://github.com/nolanlab/Rclusterpp). All clustering algorithms were performed with cells clustered on the following markers: Ki67, Mart1, HIF1a, LDH, AMPKA, p-ERK1, PFK, p-ACAC, Slug, and p-LKB. The code used for clustering is available upon request.

### Critical point analysis

Critical point analysis was implemented in two separate runs. Each run was conducted only on clusters from one of the two paths. Two quantitative indices are utilized for predicting critical transitions in a high-dimensional state space: Ic^60^ and SNAI^10^. Both of the two indices are formalized based on the mathematical features of the tipping point: increase of marker–marker correlation and increase of cellular heterogeneity. The SNAI value is defined as the reciprocal of the determinant of the protein-protein correlations. The Ic value is defined as the ratio of the average of all pairs of protein-to-protein correlation coefficients to the average of all pairs of cell-to-cell correlation coefficients and is calculated. The code used to calculate the SNAI/Ic indices for individual cell clusters is available upon request.

### Network analysis

Pair-wise correlation matrices were calculated on within each of the 14 clusters using the Hmisc R package (version 4.2-0, available from https://cran.r-project.org/web/packages/Hmisc/index.html). Spearman’s correlations were calculated. The correlation output from the Hmisc package produces the pair-wise correlation values matrix. Bonferroni corrected *p*-value was used to filter the correlation network through statistical significance and the correlation networks were drawn using a custom MATLAB code. Hub score and node degree for each marker in each correlation network were calculated using the igraph R package (version 1.2.4.1). Both scores were rescaled from 0 to 1 for each marker for side-by-side comparison and plotted to visualize marker-to-marker variation in hub behavior between methods of calculating correlation. The code used to perform the correlation network analysis is available upon request.

### mRNA extraction and qPCR

RNA was extracted from cells using the RNeasy Mini Kit or RNeasy plus Micro Kit (Qiagen) according to the manufacturer’s protocol. First-strand cDNA was synthesized from extracted total RNAs using the iScript cDNA Synthesis Kit (Bio-Rad). The expression of human Slug, MITF, MART1, and PFK transcripts were analyzed by SYBR Green-based real-time quantitative reverse-transcription PCR using specific primers (Supplementary Table 1). Data were normalized to the expression of *RPL19* and are expressed as fold changes.

### MITF-knockdown cell line

Short hairpin RNA (shRNA) targeting the coding sequence of *MITF* and control shRNA were purchased from Santa Cruz. Lentiviruses encoding control shRNA and MITF shRNA were produced in HEK-293T cells by transient transfection of lentiviral-based vectors and their packaging vectors psPAX2 and pMD2.G. The virus was collected, filtered through a 0.45 µm syringe filter after 48 h, and the M397 cells were spin-infected with viral supernatant supplemented with 10 µg/mL polybrene at 900 g and 30 °C for 90 min. The transduced cells were selected using puromycin, starting at 3 days post transduction.

### MITF-reporter cell line

The human *Tyrosinase Promoter* was subcloned from pLightSwitch Prom S700747 (SwitchGear Genomics, Carlsbad, CA) into the BamH1 and EcoRI sites of the lentiviral vector backbone, driving the expression of the *Zsgreen* gene. Lentivirus particles were generated as described above to stably transduced M397 cells. A clonal cell line was further generated via single-cell sorting and expansion. Cells were then sorted as GFP-High and GFP-Low population by BD FACSAria Fusion Cell Sorter for further treatment and analysis.

### Fluorescence microscopy

Images were acquired at ×10 (Olympus, 10X FL PH, 0.3 NA) on an EVOS FL Auto Imaging System (Fisher Scientific) in Yellow fluorescent protein (YFP) and DIC channels. Light or laser intensity, exposure, and gain were set to be the same between MITF-High well and MITF-Low well.

### Flow cytometry analysis

All cells were then fixed with Fix-Perm buffer from BD Bioscience and then stained for intracellular dye-conjugated antibodies for MART1, NGFR, AXL, p-ERK, and Ki67. Flow cytometry analysis was conducted using Attune NxT Flow Cytometer from Thermo Fisher and the data were analyzed using FlowJo software. To visualize the cell-state transition trajectories, the six-dimensional flow cytometry data were projected onto a 3D and 2D space via surprisal analysis and t-SNE, respectively.

### Fluorescence-activated cell sorting

Cells were washed and trypsinized from culture plates, following by centrifugation at 500 × *g* and 4 °C for 5 min to pellet cells. Cell pellets were then resuspended in PBS containing 1% BSbovine serum albumin before fluorescence-activated cell sorting. The gating strategy is shown in Supplementary Fig 29.

### Senescence associated β-galactosidase activity

Percentage of the senescent cell was quantified using the Senescence β-Galactosidase Staining Kit (Cell Signaling, 9860) according to the manufacturer’s protocol. Briefly, cells on the plate were rinsed with PBS and then fixed with the Fixative Solution for 15 min. After fixation, the plate was rinsed with PBS two times and then incubated at 37 °C overnight in staining solution. Plates were examined under phase-contrast microscopy.

### Apoptosis assays

Cell apoptosis assays were performed by treating indicated cell lines cultured under respective conditions. Cells were stained with Annexin V–fluorescein isothiocyanate and propidium iodide for 15 min at room temperature before flow cytometry analysis. Gates were determined using an unstained control.

### Clonogenic assay

M397 cells were plated onto six-well plates with fresh media at an optimal confluence. The media (with drug or DMSO) were replenished every two days. Upon the time of staining, 4% paraformaldehyde was applied onto colonies to fix the cells and a 0.05% crystal violet solution was used for staining the colonies.

### Reporting summary

Further information on research design is available in the Nature Research Reporting Summary linked to this article.

## Supplementary information

Supplementary Information

Reporting Summary

## Data Availability

All the data supporting the findings of this study are available within the article and its Supplementary Information files, and from the corresponding author upon reasonable request. Raw data for underlying Figs. 3c–f, h–i and 5a–e, and Supplementary Figs. 3, 4, 5, 6, and 21a–c are provided in the Source Data file.

## Source Data

Source Data

## Code availability

Custom code for analysis of raw CSV output of the SCBC measurements was written in R and has been made available/open-source via GitHub (https://github.com/mesako/Melanoma-Publication). The code used to generate the exact FLOW-MAP graphs, the single-cell clustering, the calculation of SNAI/Ic indices for individual cell clusters, and the correlation network analysis is available upon request.
