# Supplementary material for: Multi-omic single-cell snapshots reveal multiple independent trajectories to drug tolerance in a melanoma cell line
Source: Nat Commun. 2020 May 11;11:2345. doi: 10.1038/s41467-020-15956-9 (PMC7214418; doi:10.1038/s41467-020-15956-9)
Supplement: Supplementary file 1 — Supplementary Information [file 41467_2020_15956_MOESM1_ESM.pdf]

Supplementary Information

Multi-omic single-cell snapshots reveal multiple independent trajectories to drug tolerance in a melanoma cell line

Su *et al*

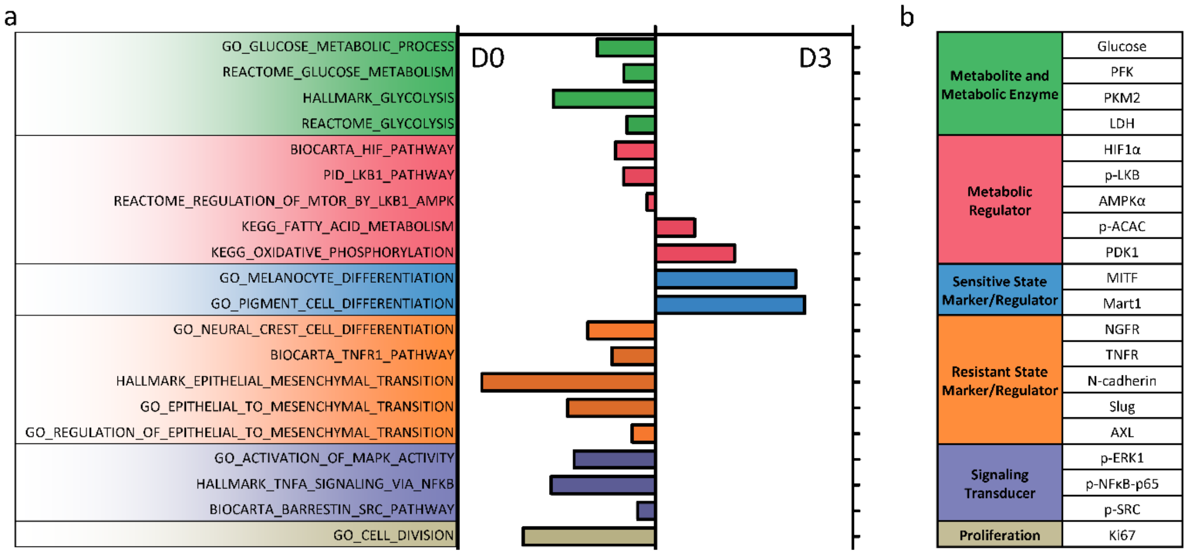

Supplementary Figure 1. Transcriptomic analysis guides the selection of panel markers. a. Pathways that are differentially altered from day 0 to day 3 after BRAFi treatment. Each row represents a certain signaling pathway and each bar indicates normalized enrichment score (NES) calculated from geneset enrichment analysis (GSEA) of cells harvested at day 3 versus day 0. Each pathway is color-coded by its functional category as described in Fig. S1b. **b.** Panel of markers per pathway selected to quantify with single-cell barcode chip (SCBC) analysis. 20 markers were selected for SCBC analysis. Markers with similar biological function are organized together and color-coded by functional category.

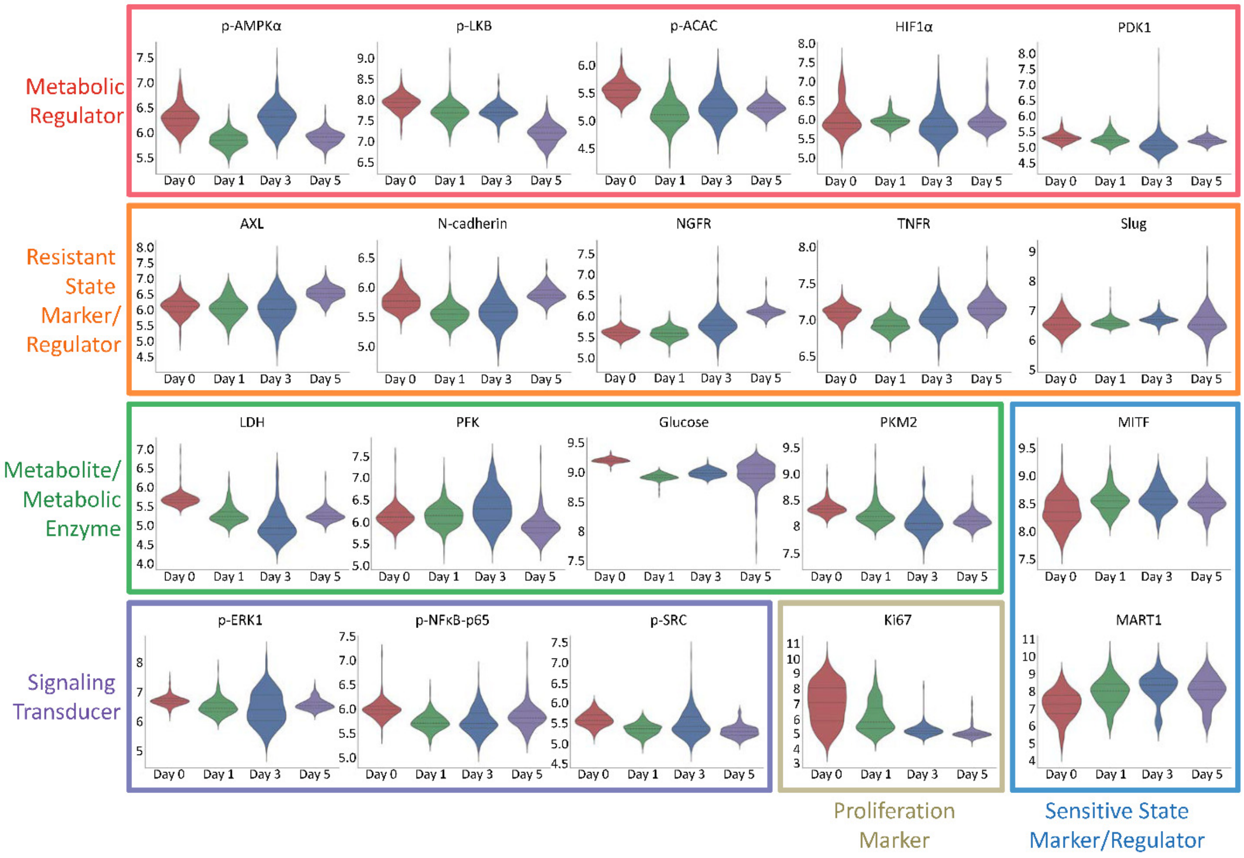

Supplementary Figure 2. Distribution of all 20 markers across 4 time points. Each of the 20 plots represents the distributions of a certain marker level across 4 time points. Y-axis represents natural log of measured marker level. Markers within the same functional category are boxed together. Border color of each plot corresponds to the functional category of each marker, as described in Fig. 1a.

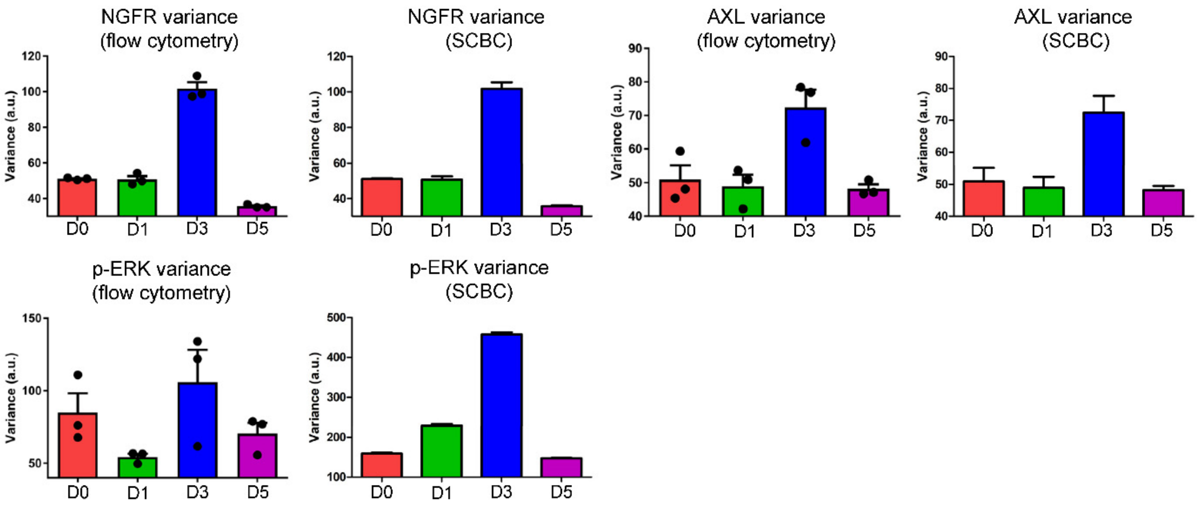

Supplementary Figure 3. Quantitation of variance of three different markers during BRAFi treatment. Y-axis represent the variance of the marker-level distribution. Data are presented as mean values +/- SEM. Plots for flow cytometry experiments is the result of n = 3 biologically independent samples per group. For SCBC dataset, N=100 independent bootstrap runs are performed. Source data are provided as a Source Data file.

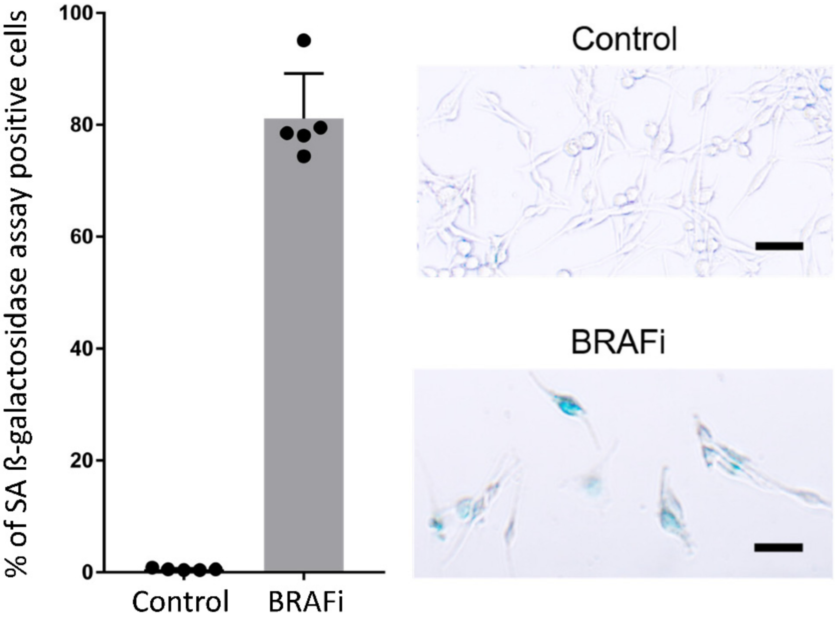

Supplementary Figure 4. SA ß-galactosidase assay of untreated control and BRAFi-treated cells. Left panel: Significant increased percentage of SA ß-galactosidase positive cells was observed in BRAFi treated day5 cells. Data are presented as mean values +/- SEM. Right panel: representative image of untreated melanoma cell and cells treated with BRAFi for 5 days. Scale bar 50um. Each experiment is the result of n = 5 biologically independent samples per group. Source data are provided as a Source Data file.

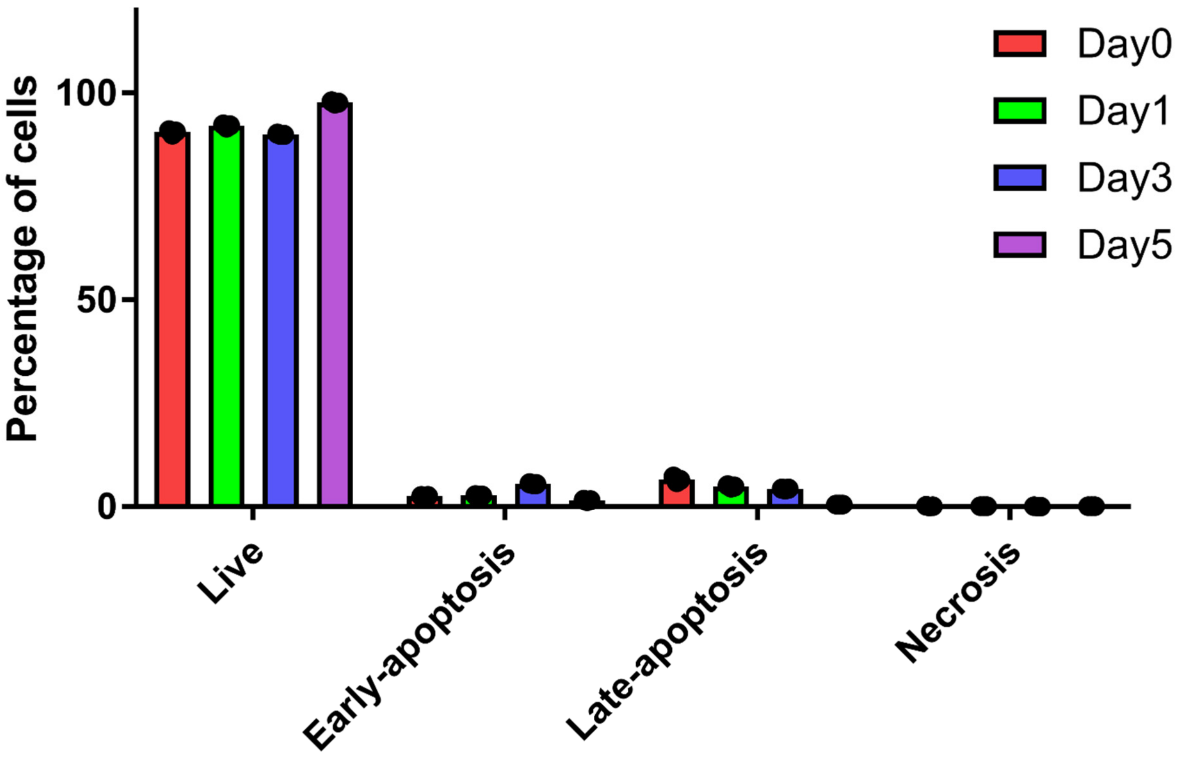

Supplementary Figure 5. Percentage of apoptosis cells across five days of BRAFi treatment. Data are presented as mean values +/- SD. Each experiment is the result of n = 3 biologically independent samples per group. Source data are provided as a Source Data file.

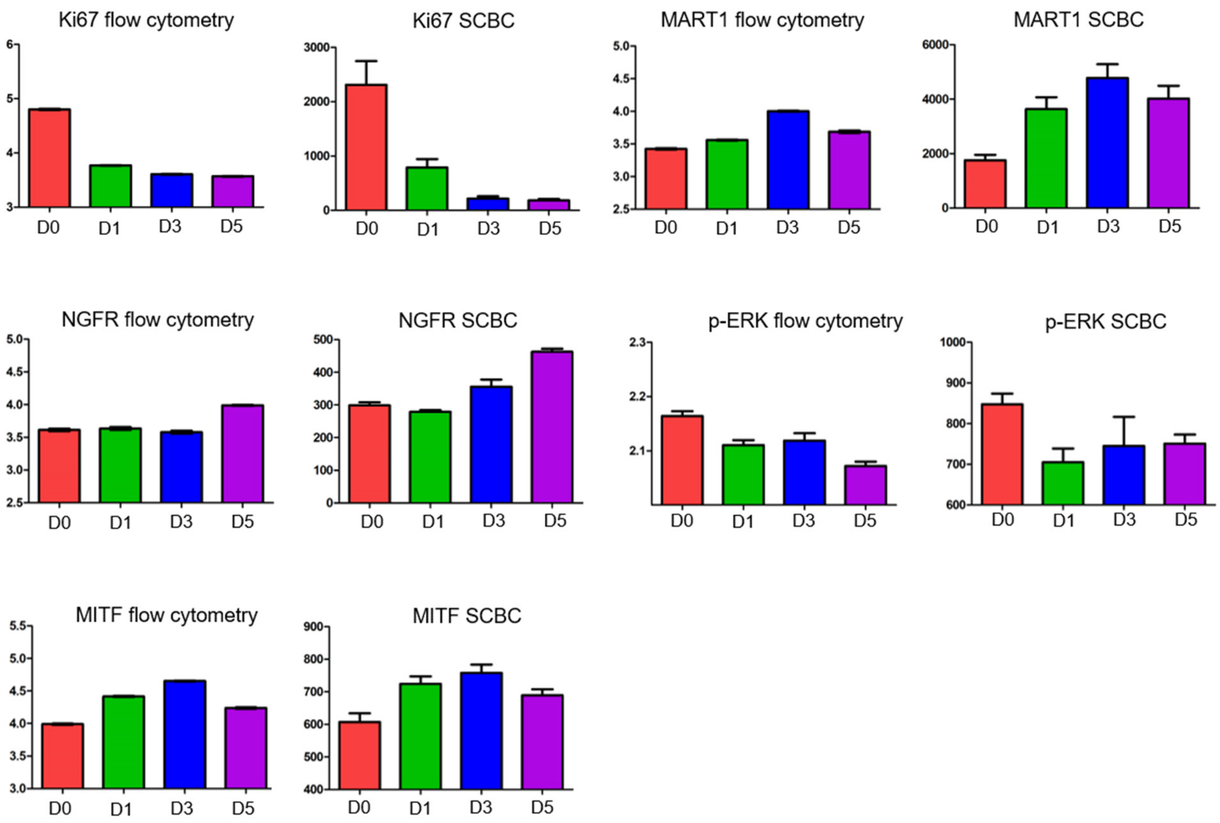

Supplementary Figure 6. Comparison of Flow cytometry analysis and SCBC data. X-axis represent different time points after drug treatment. Y-axis of the flow cytometry data represent the log of measured fluorescent intensity. Y-axis of the SCBC data represent measured level of the respective markers. Error bars represent 95% confidence interval of the data and the center represents mean value. For SCBC, N= 156, 185, 162 and 171 single cells are independently analyzed for day0, day1 day3 and day5 respectively. Source data are provided as a Source Data file.

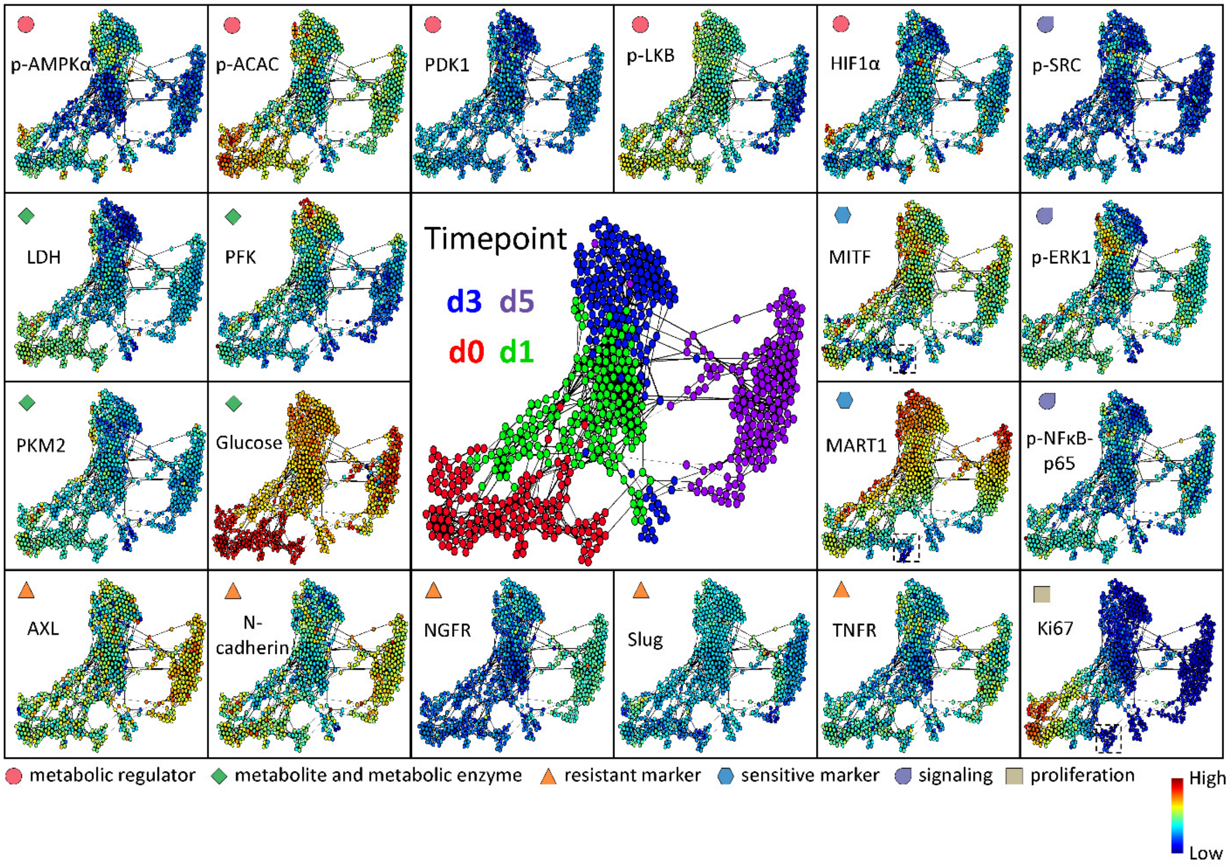

Supplementary Figure 7. Visualization of SCBC data by FLOW-MAP. Each dot represents an individual cell. The distance between each pair of cells represents the overall multi-omic dissimilarity between them. Cell pairs that are close enough are linked with an edge in between. The colors of the dots in the central panel represent BRAFi exposure time (0, 1, 3, or 5 days) of the corresponding cells. Dot colors in the other panels represent the abundance of each marker in each cell. Markers belonging to the same functional category, as described in the bottom of the figure, were assigned to a certain shape and color. The dashed-line box in the panels for MITF, MART1, and Ki67 levels shows a small subpopulation of day-0 cells that are slow cycling with less melanocytic phenotype.

.

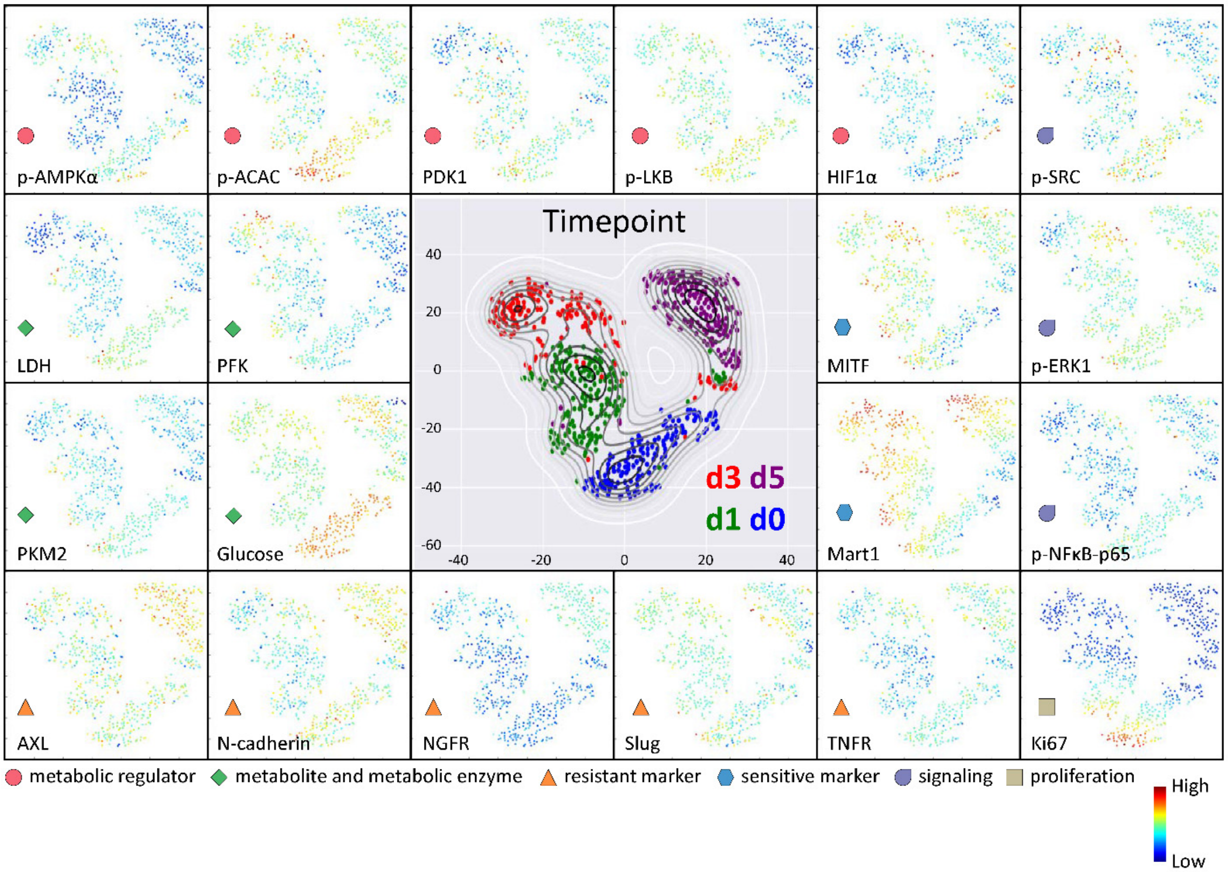

Supplementary Figure 8. Visualization of marker abundance by t-SNE. Each dot per plot represents an individual cell. The distance between each pair of dots represents the overall multi-omic dissimilarity between that pair of cells. The dot colors in the central panel represent the drug exposure time of each cell. Dot colors in the other panels represent the abundance of the specified marker in each cell. Markers that belong to the same functional category were assigned to a certain shape and color, as described in the bottom of the figure. T-SNE visualizations show both the heterogeneity that exists at baseline as well as the progression across time through two separate paths.

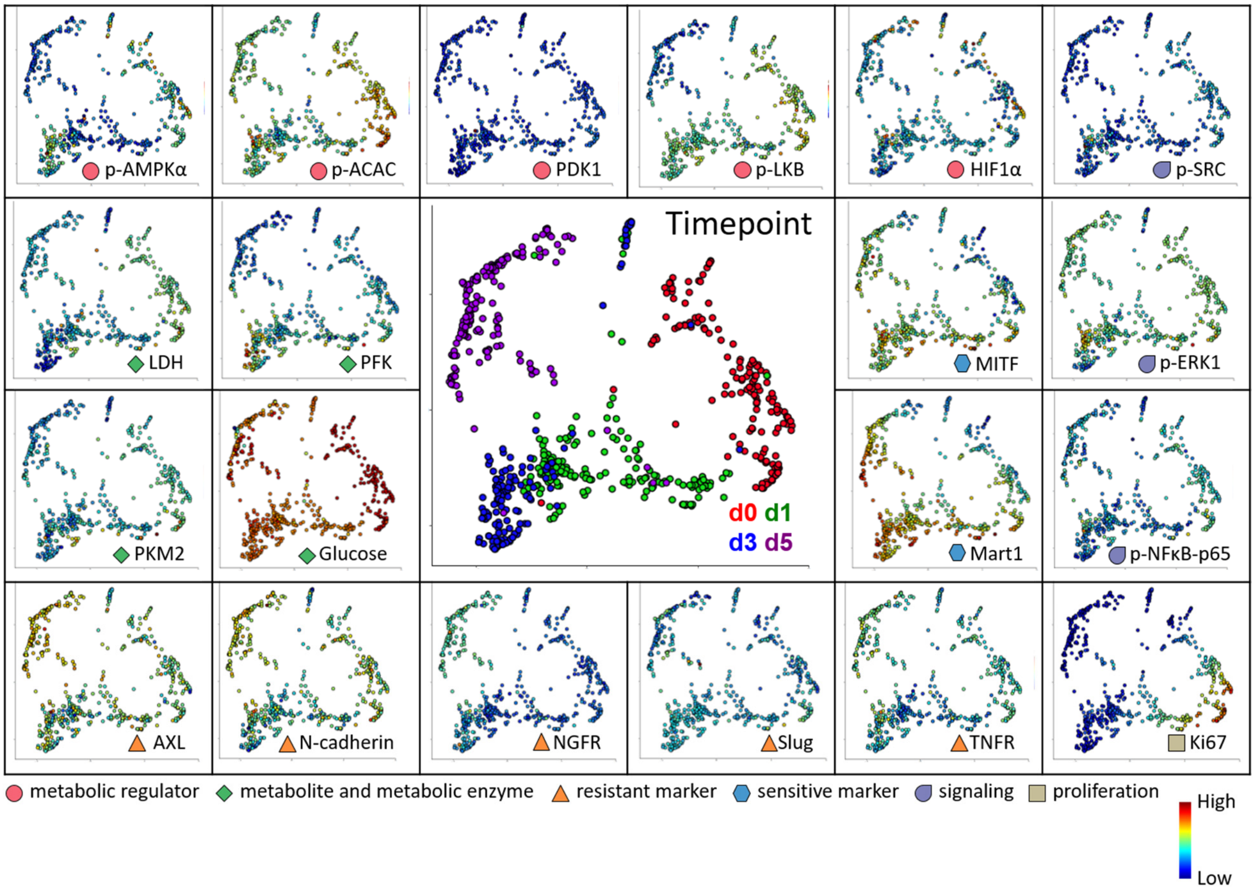

Supplementary Figure 9. Visualization of marker abundance by PHATE. Each dot per plot represents an individual cell. The distance between each pair of dots represents the overall multi-omic dissimilarity between that pair of cells. The dot colors in the central panel represent the drug exposure time of each cell. Dot colors in the other panels represent the abundance of the specified marker in each cell. Markers that belong to the same functional category were assigned to a certain shape and color, as described in the bottom of the figure. PHATE visualizations show both the heterogeneity that exists at baseline as well as the progression across time through two separate paths.

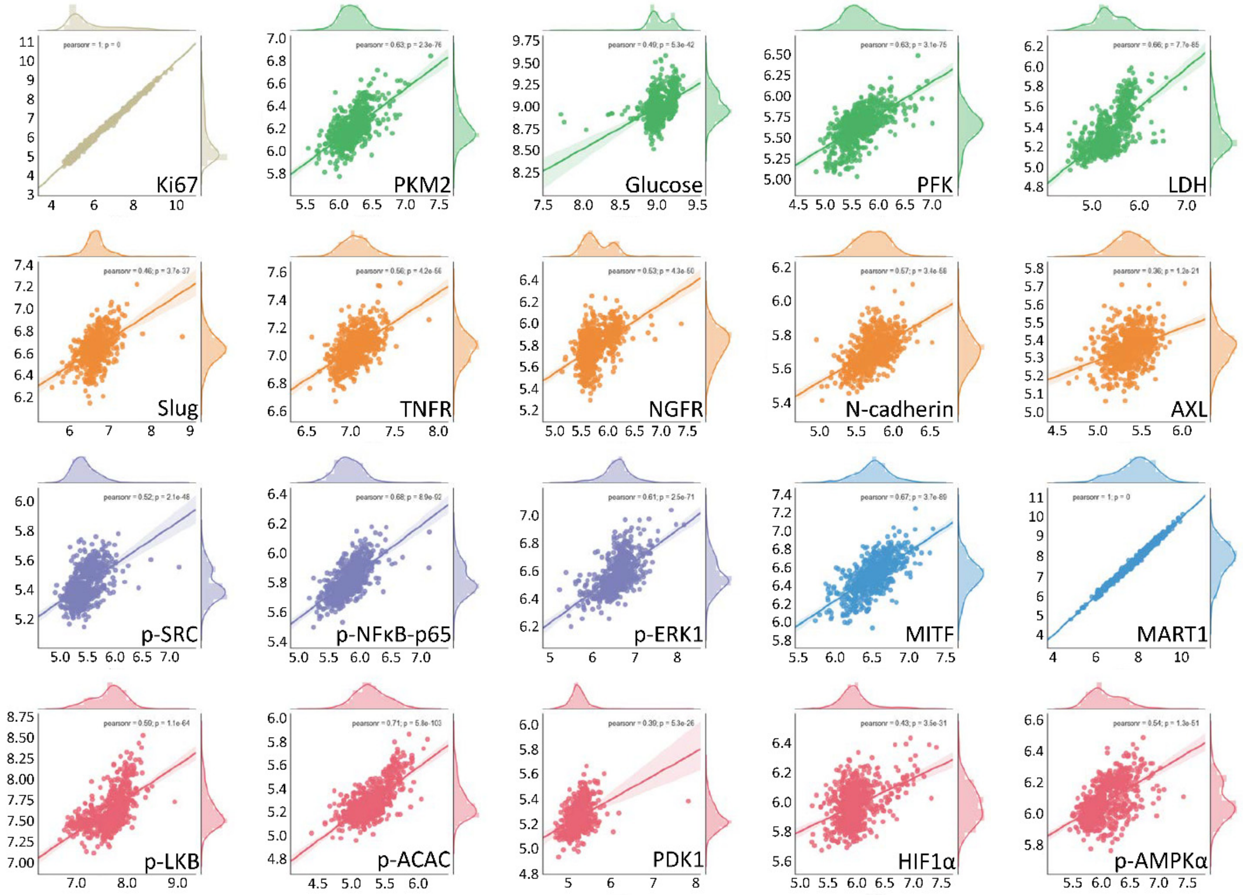

Supplementary Figure 10. Two modules from surprisal analysis recapitulates the experimental data. Each plot represents an individual marker. Each dot within a single plot represents a single cell. The x-axis value of each dot represents the experimentally measured marker expression within a cell. The y-axis value of each dot represents the predicted marker level of the same cell as calculated by surprisal analysis of only module1 and module2. The strong positive correlation between the x- and y-axis values indicate that surprisal analysis of the two modules recapitulates experimentally measured marker levels per cell. Linear regression with Pearson correlation r and two-tailed P value, n = 674. No adjustments were made for multiple comparisons.

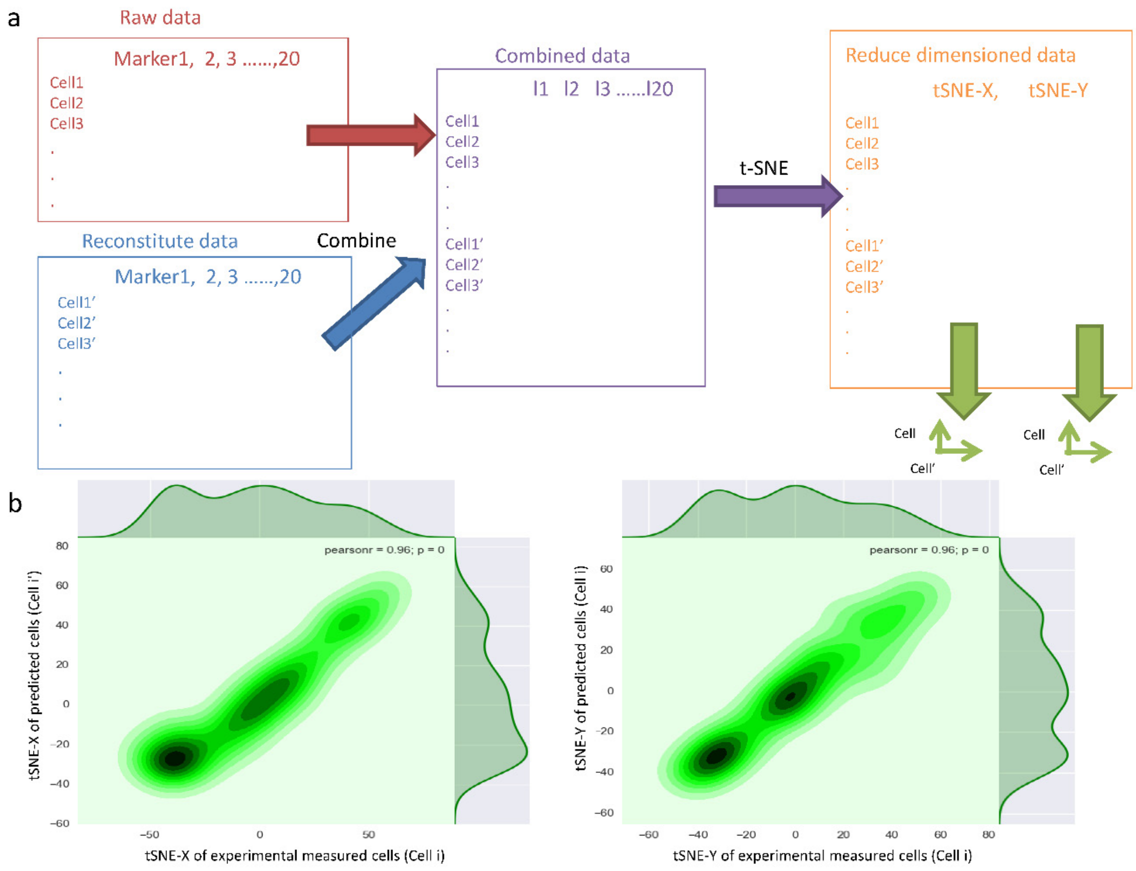

Supplementary Figure 11. Two modules from surprisal analysis recapitulates the experimental data. a. Schematic illustration of workflow to project raw data and surprisal analysis-predicted data onto the same 2-dimensional space. Each cell has measured levels of all 20 markers. Similarly, each cell also has predicted levels of all 20 markers as calculated from surprisal analysis. The raw and surprisal-predicted data matrices were combined to make a bigger matrix with double the original number of rows, each row representing a cell from raw data or predicted data. Each column represents a single marker, with each matrix value representing a single cell's abundance of a marker. The combined, 20-dimensional dataset was projected onto a single t-SNE map where cells with similar levels of all 20 markers will be in nearby coordinates. **b.** Each dot represents an individual cell. In the left panel, the x-axis represents the t-SNE x-value of the cell projected from raw data, while the y-axis represents the t-SNE x-value of the cell projected from surprisal analysis-predicted data. The right panel is similar to left panel, but instead compared t-SNE y-values. The linear, x = y plots indicate that single cells, as projected from raw data and from surprisal analysis-predicted data, are in the same location in a reduced dimension; therefore, the experimentally measured and surprisal analysis-predicted expression profiles of all 20 markers are similar.

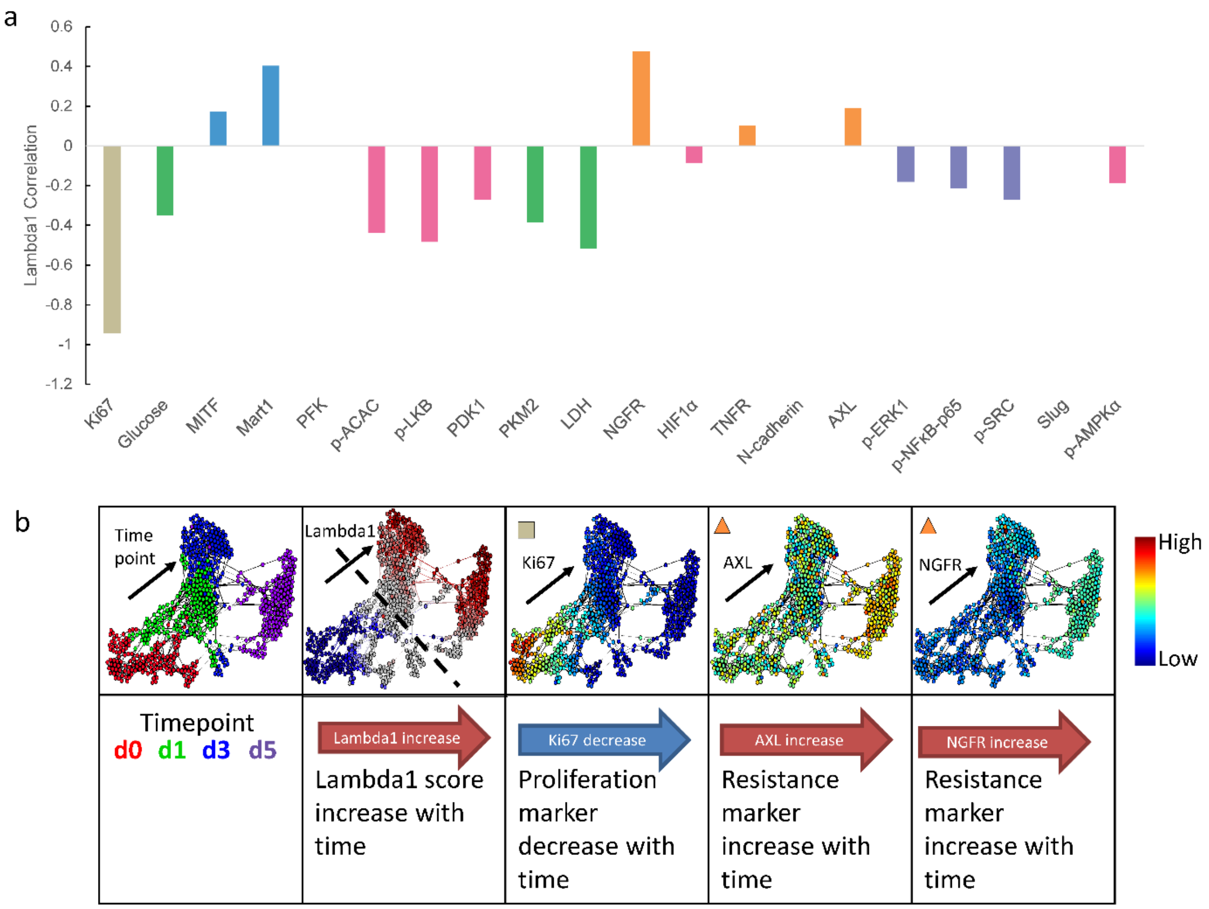

Supplementary Figure 12. Lambda1-associated markers displays time-dependent changes. **a.** Pearson correlation of marker level vs. module1 score (lambda1) across cells from all timepoints of BRAFi exposure. Correlations that are not statistically significant (i.e. $p > 0.05$) are not shown. **b.** Representative markers that showed strongest positive (AXL, NGFR) or negative (Ki67) correlation with module1 score are shown in individual cells on FLOW-MAP.

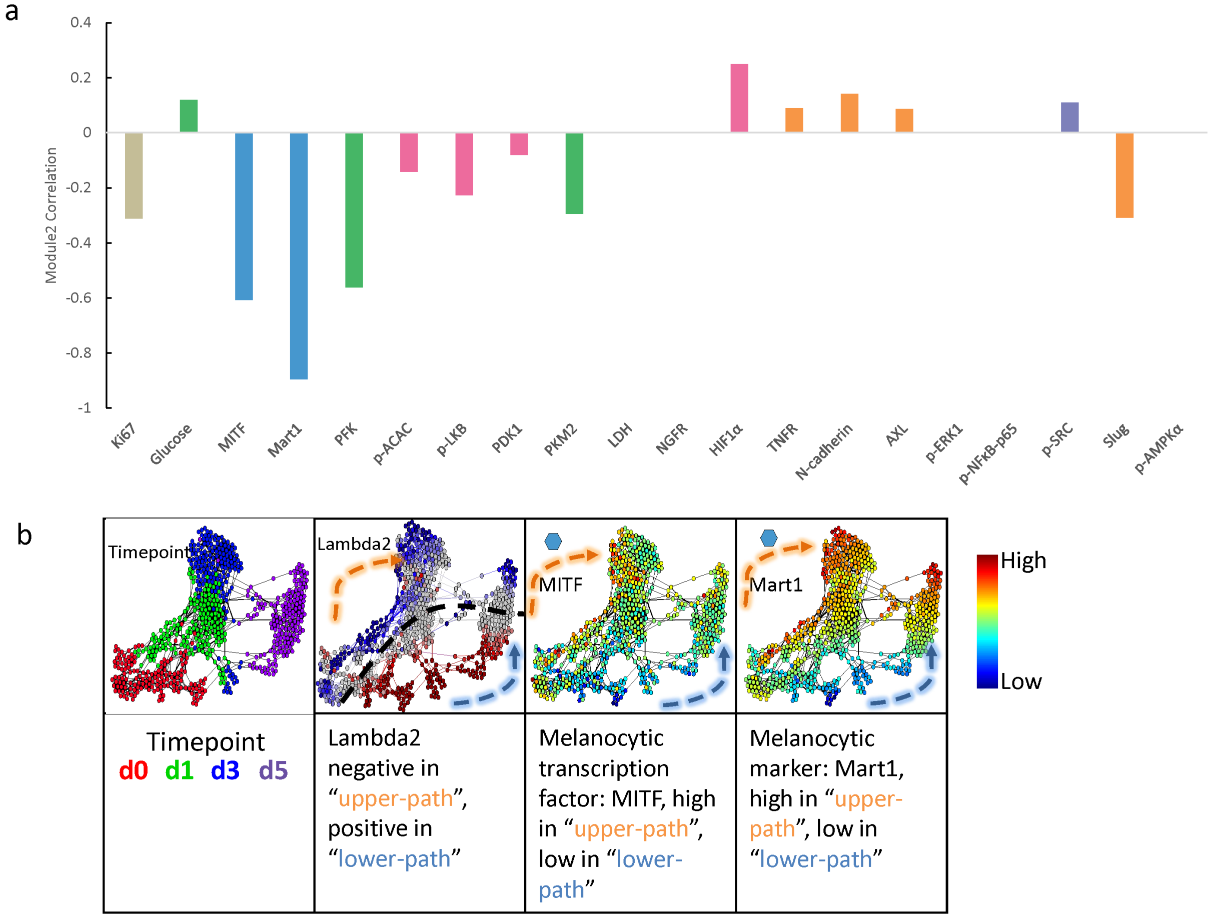

Supplementary Figure 13. Lambda2-associated markers displays path-specific expression patterns.

a. Pearson correlation of marker level with module2 score (lambda2) across cells from all time points after BRAFi exposure. Correlations that are not statistically significant (i.e. $p > 0.05$) were not shown.**b.** Representative markers that showed strongest negative correlation with module1 score are shown in individual cells on FLOW-MAP.

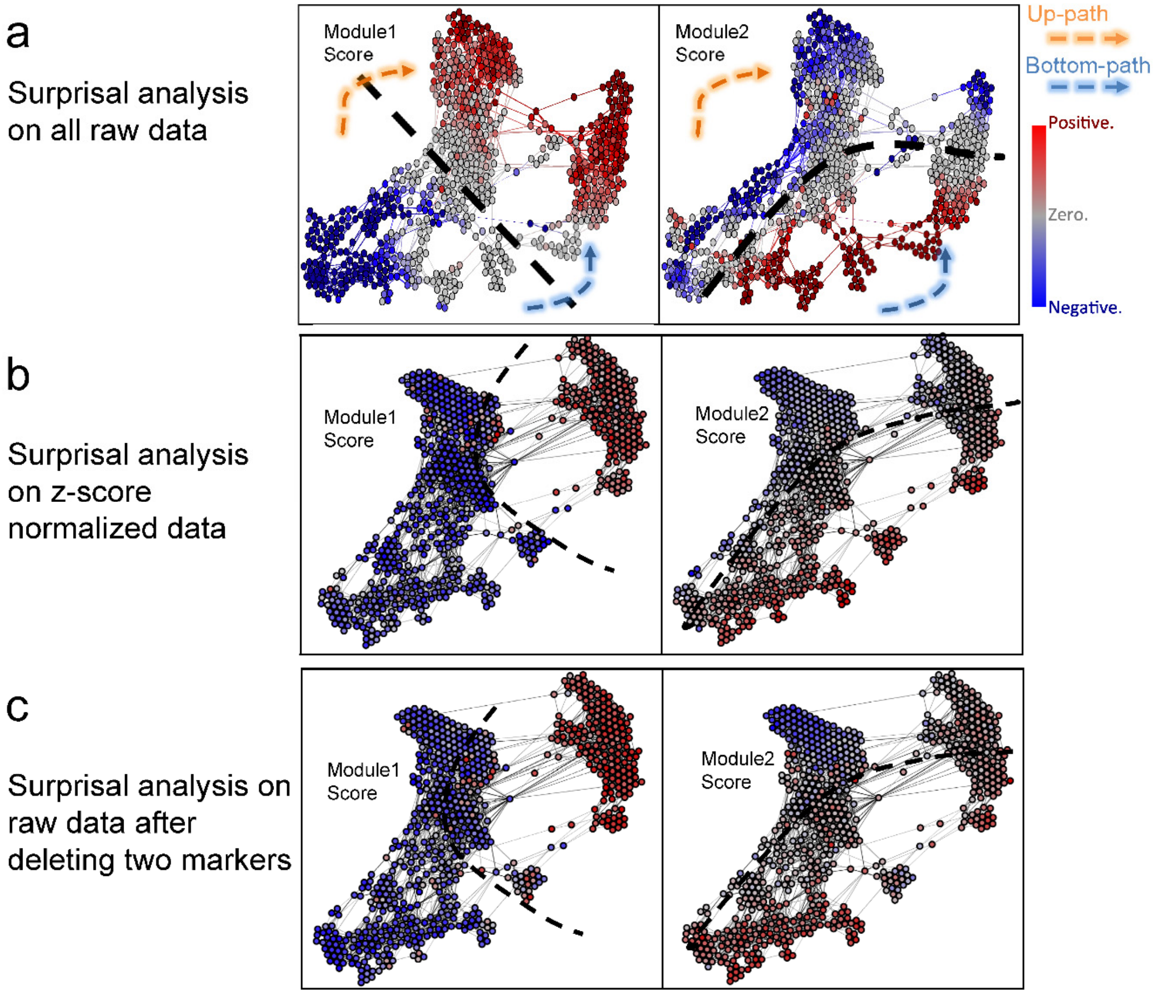

Supplementary Figure 14. Comparison of different surprisal analysis methods. a. Surprisal analysis on all raw data. **b.** Surprisal analysis based on z-score normalized data. **c.** Surprisal analysis on subset of data after deleting top two most variable markers: KI67 and MART1.

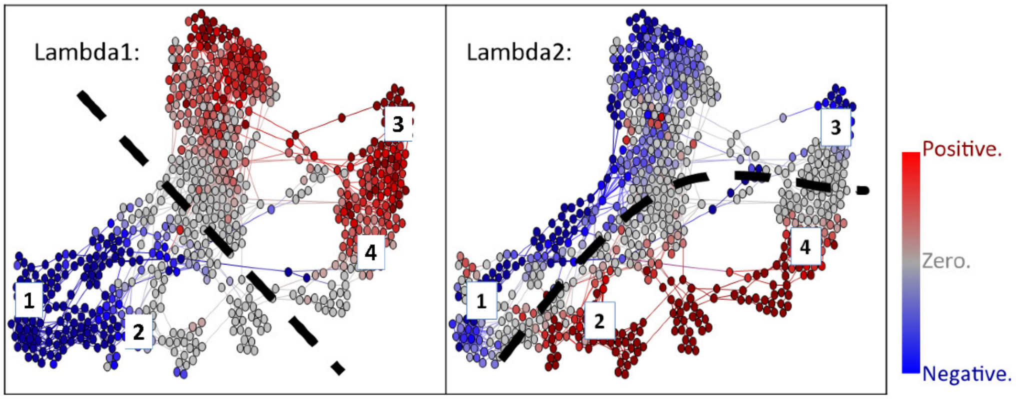

Supplementary Figure 15. Identification of Module1- and Module2-associated biophysical barriers. Module1 and module2-associated barriers, as defined by the points at which a module score changes sign, separate the cells into roughly 4 different states, labeled from 1 to 4. States 1 and 2 are separated from states 3 and 4 by the module1-associated barrier. States 1 and 3 are separated from states 2 and 4 by the module2-associated barrier.

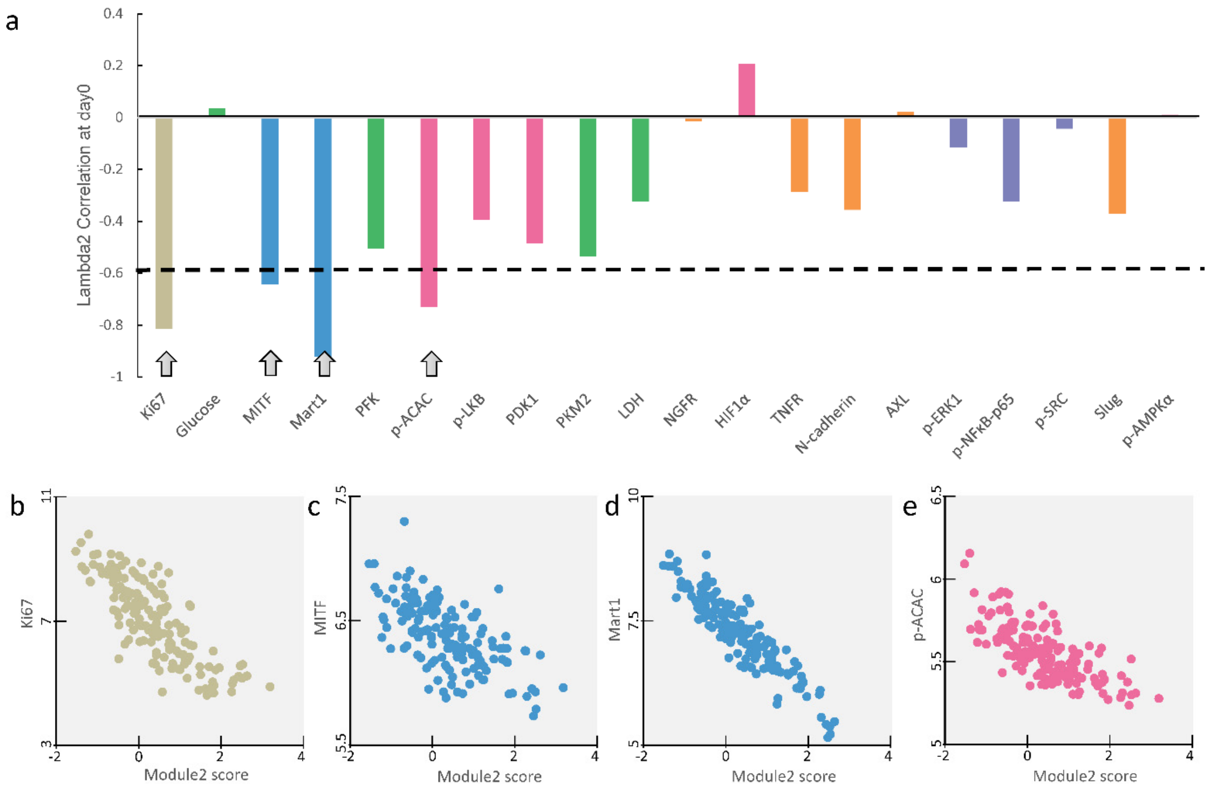

Supplementary Figure 16. Correlation analysis of markers with module2. a. Pearson correlation of marker level and module2 score in day 0 cells from single-cell dataset. The four most highly-correlated markers are labeled with gray arrows. **b.** Scatter plots showing expression levels of the four most highly-correlated markers versus module2 score in day-0 cells from single-cell dataset.

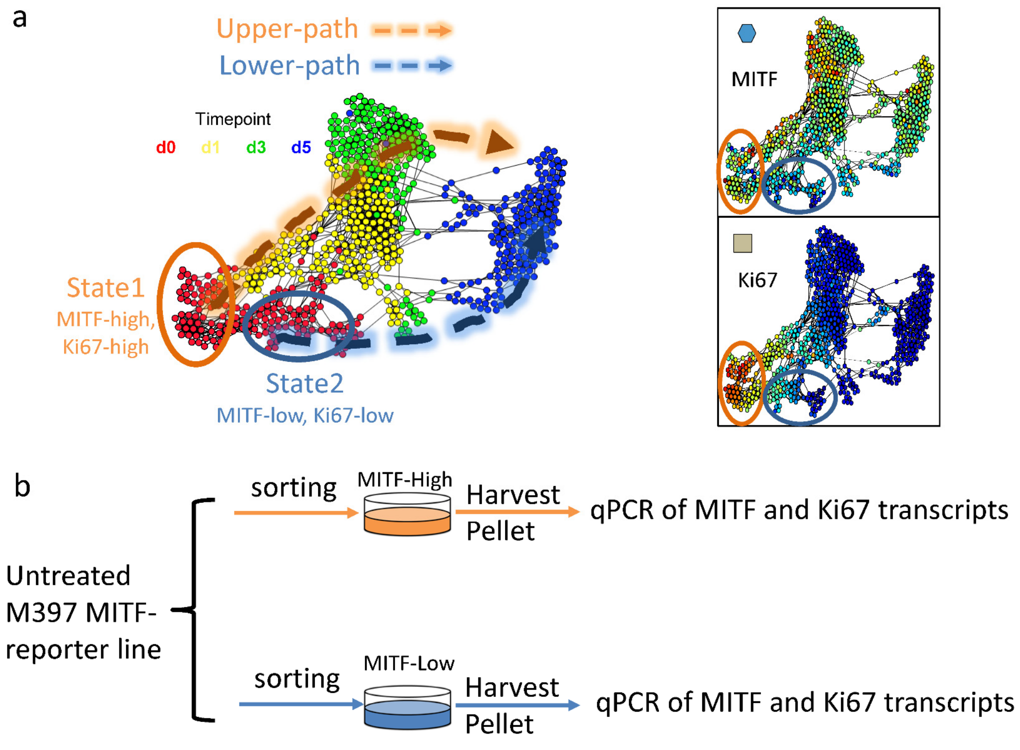

Supplementary Figure 17. Illustration of assays using untreated MITF-reporter cells. a. Untreated cells in state 1 and state 2 showed significantly different levels of MITF and Ki67. **b.** For MITF-GFP reporter line, cells with higher GFP level and lower GFP level were sorted out using FACS. The sorted cells were then harvested for qPCR quantitation of MITF and Ki67 expression.

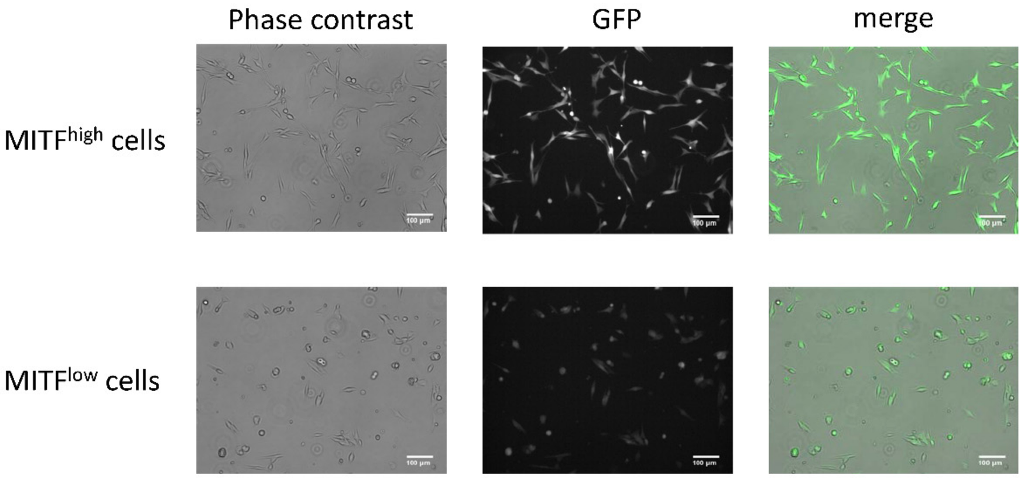

Supplementary Figure 18. MITF-GFP level and morphology of cells. Representative images from three biological replicates are shown.

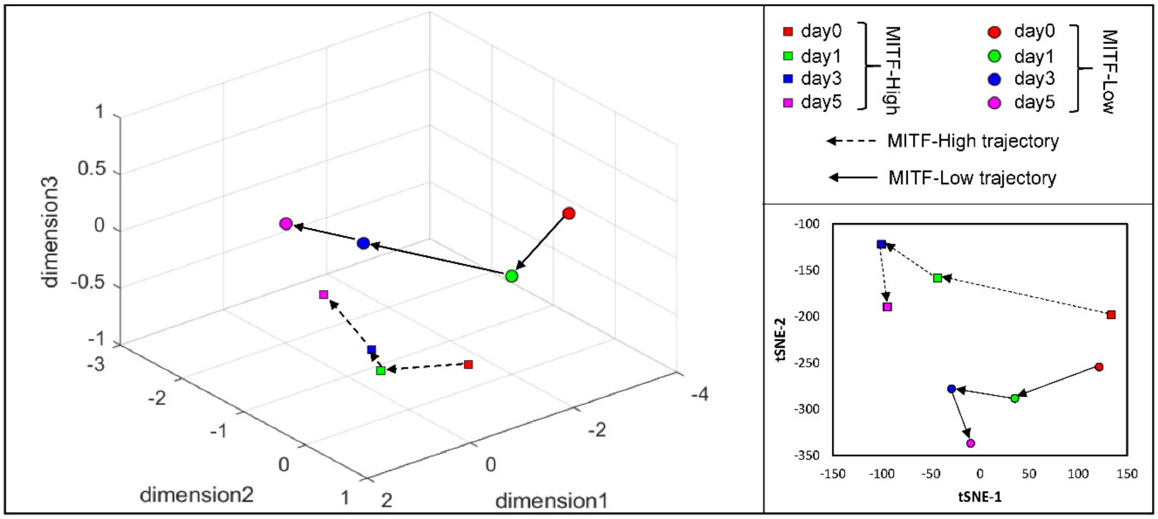

Supplementary Figure 19. Visualization of the trajectories of the MITF-High and MITF-Low subpopulations. Six key markers from flow cytometry assays are analyzed via three-dimensional visualization. Each dot represent certain subpopulation analyzed at certain time point. Samples were projected from the original 6-dimensional space onto a 3D (left panel) or 2D (bottom right panel) space as a single dot.

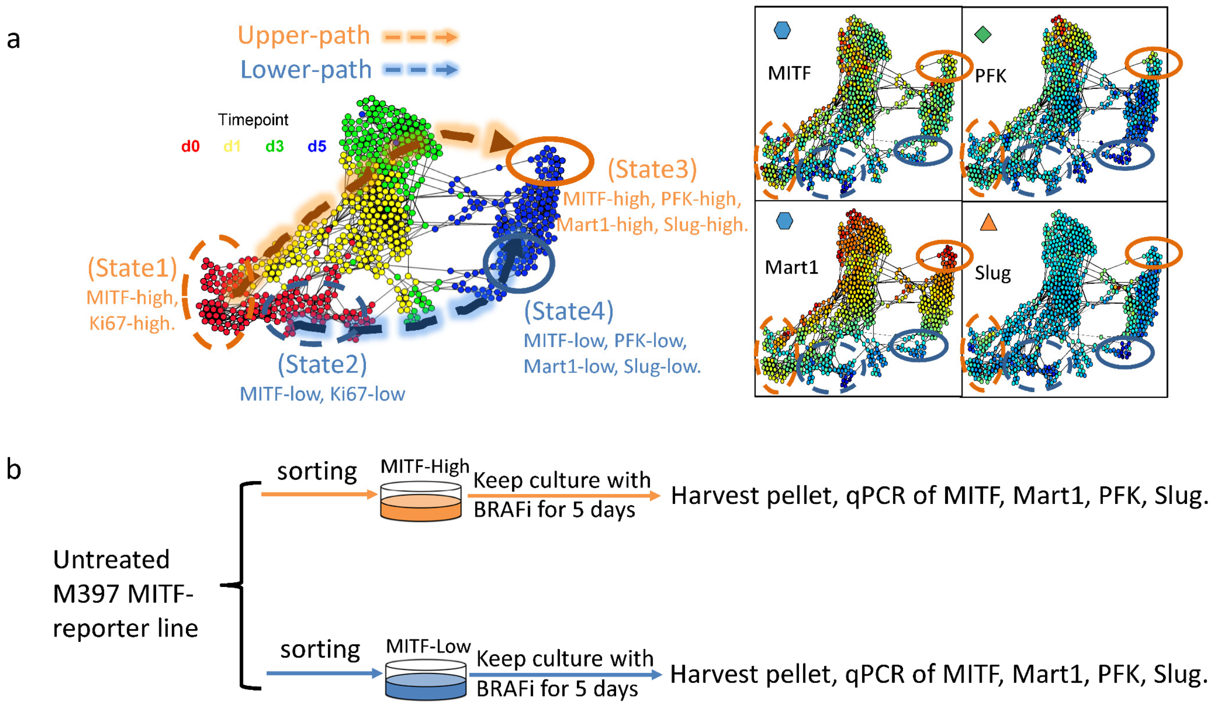

Supplementary Figure 20. Illustration of assays using BRAFi-treated MITF-reporter cells. a. Day-5 cells in state 3 and state 4 showed different levels of MITF, MART1, PFK and Slug. **b.** For MITF-GFP reporter line, cells with higher GFP level and lower GFP level were sorted out using FACS. The sorted cells were then treated with BRAFi for another five days, then harvested for qPCR quantitation of MITF, MART1, PFK and Slug expression.

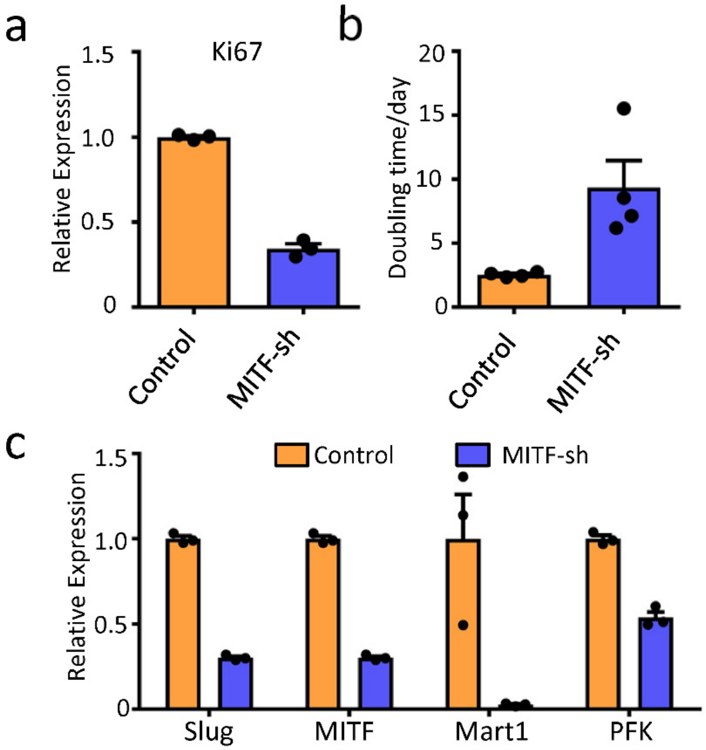

Supplementary Figure 21. MITF KD cells shows similar phenotype as state 2 cells. a. Expression level of Ki67 from qPCR of MITF knockdown cells versus control cells. Each experiment is the result of n = 3 biologically independent samples per group. **b.** Measured doubling time of MITF-knockdown cells versus control cells. Each experiment is the result of n = 4 biologically independent samples per group. **c.** Expression level of MITF, MART1, PFK and Slug after 5 days of BRAFi treatment in control cells and MITF-knockdown cells. Each experiment is the result of n = 3 biologically independent samples per group. Data are presented as mean values +/- SEM. Source data are provided as a Source Data file.

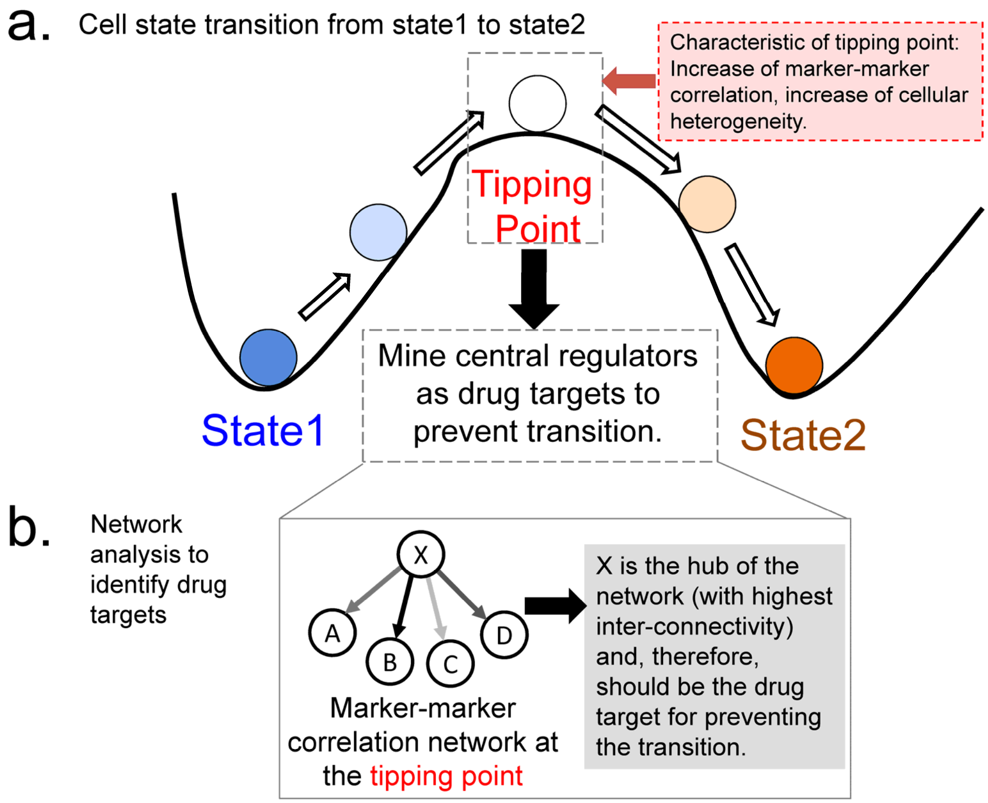

Supplementary Figure 22. Illustration of cell state transition and network analysis. a. Cells transit from state1 to state2, passing over a barrier, the peak of which is the critical (tipping) point. The barrier is not directly measured. Instead, the tipping point is identified based on quantitative characteristics extracted from the single cell assays, such as increased marker-marker correlations, that are generally characteristic of critical points. **b.** Network analysis of tipping point marker correlations to identify network hubs, is a strategy to identify effective drug targets for preventing the transition.

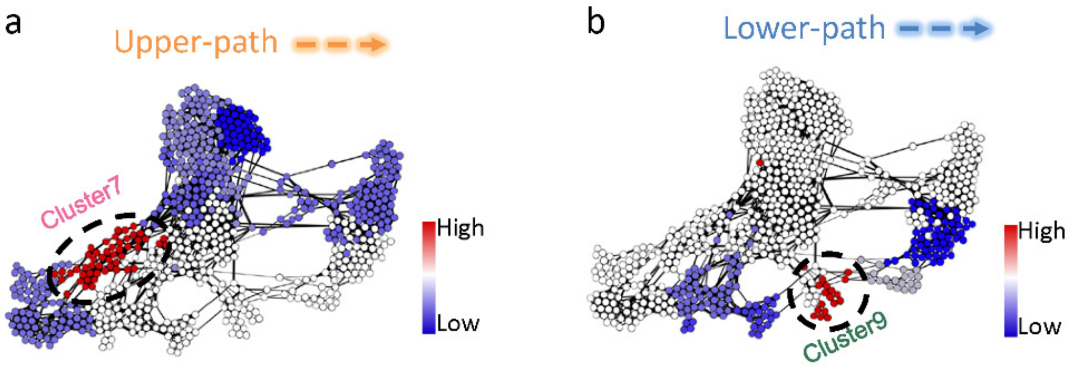

Supplementary Figure 23. Critical point transition analysis of each trajectory. **a.** Critical point transition analysis for upper path. Critical point index Ic is calculated within each subpopulation associated with the upper path and color-coded onto the FLOW-MAP. Red indicates higher Ic value. Blue represents lower Ic value. Cluster 7, circled and labeled, shows the highest Ic value in the upper path. **b.** Critical point transition analysis for lower path. Critical point index Ic is calculated within each subpopulation associated with the lower path and color-coded onto the FLOW-MAP. Red indicates higher Ic value. Blue represents lower Ic value. Cluster 9, circled and labeled, shows the highest Ic value in the lower path.

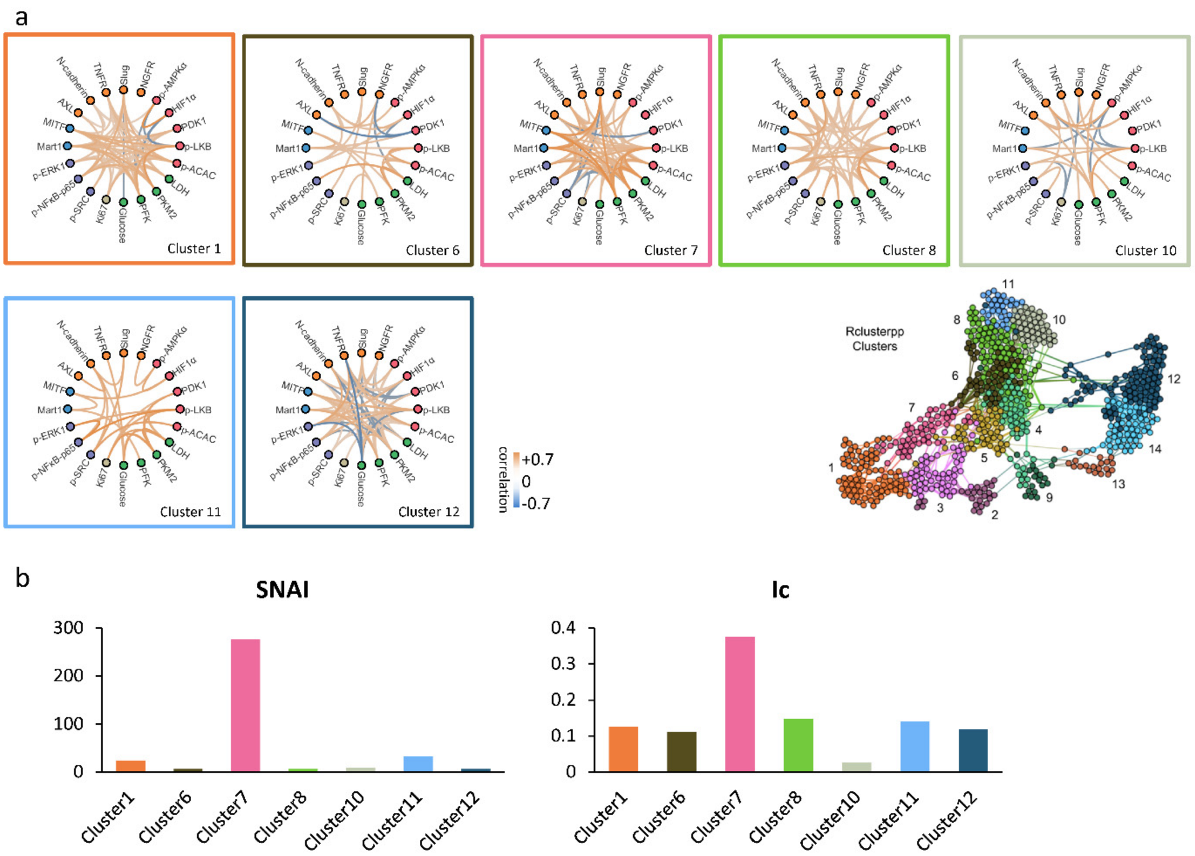

Supplementary Figure 24. Network structure and SNAI/Ic values for subpopulations in the upper path. a. Network of subpopulations associated with the upper path. Each network structure plot is bordered by the color label of the corresponding cluster. **b.** SNAI and Ic values of networks associated with subpopulations in the upper path.

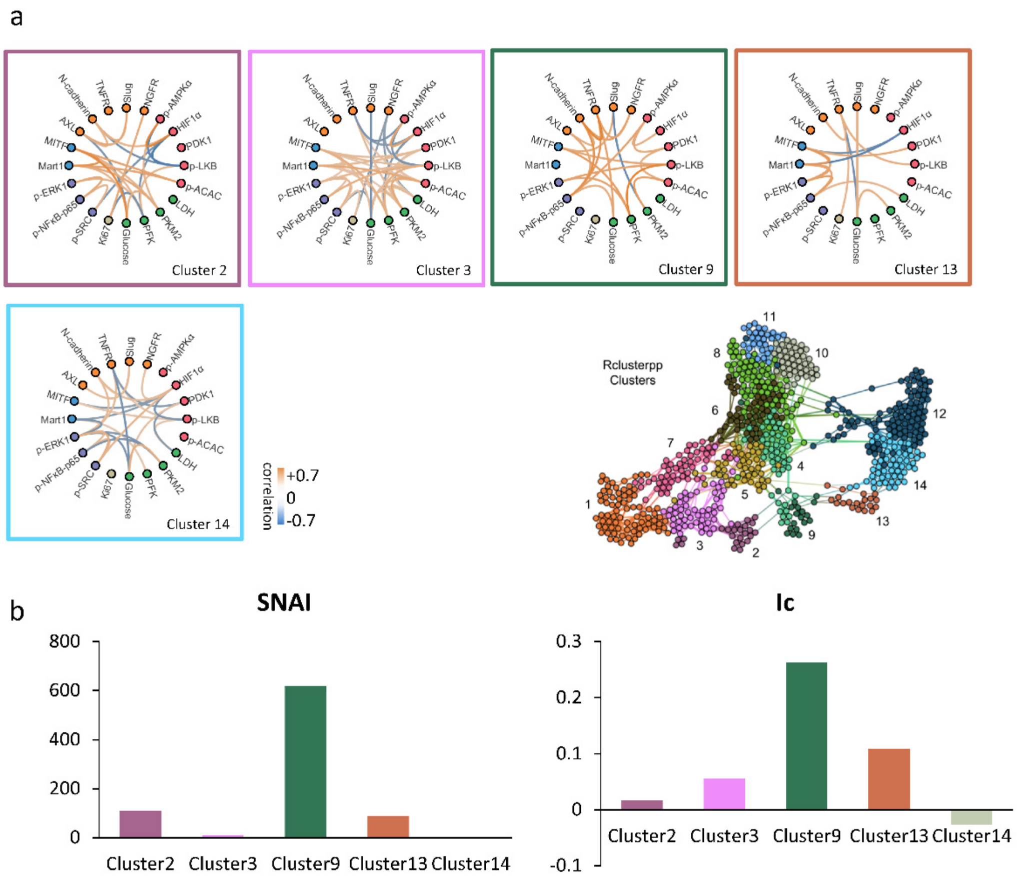

Supplementary Figure 25. Network structure and SNAI/Ic values for subpopulations in the lower path. a. Network of subpopulations associated with the lower path. Each network structure plot is bordered by the color label of the corresponding cluster. **b.** SNAI and Ic values of networks associated with subpopulations in the lower path.

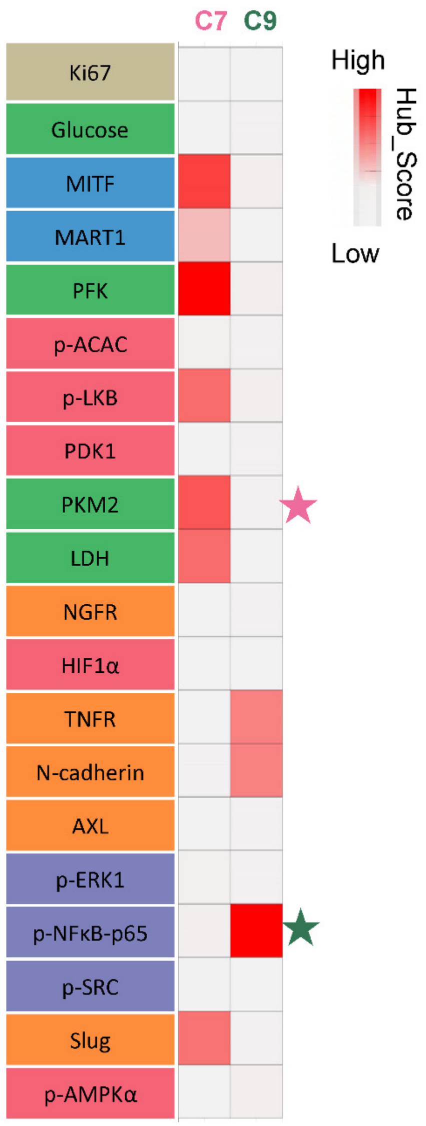

Supplementary Figure 26. Hub-score of cluster7 (C7) and cluster9 (C9). Colors in C7 and C9 columns indicate the hub-score value of each node found within the cluster 7 or cluster 9 networks, respectively. Nodes labeled with stars were further tested using drug perturbation.

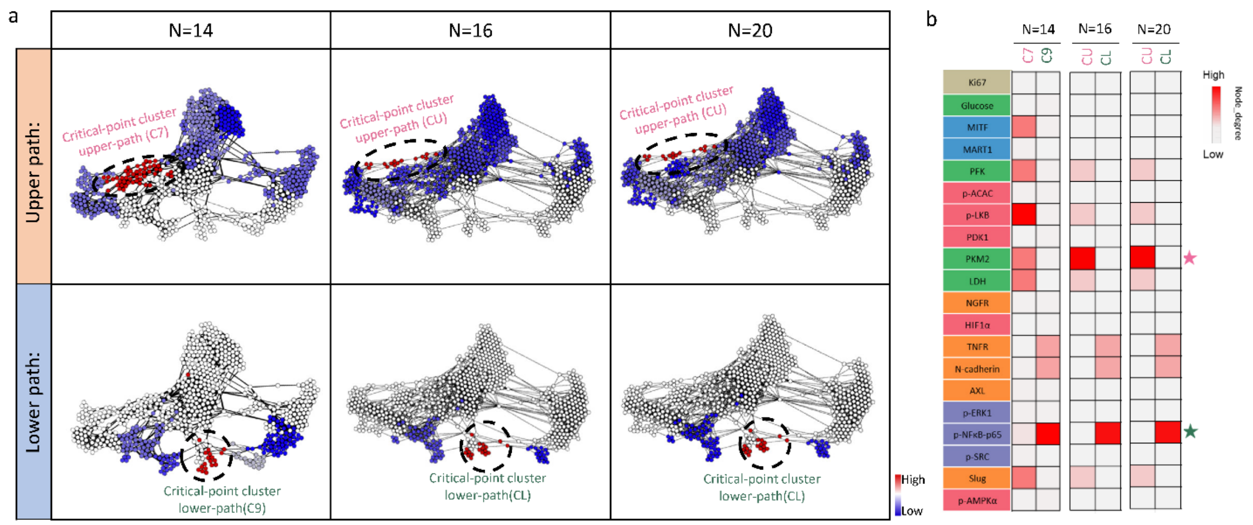

Supplementary Figure 27. Critical point analysis across different numbers of clusters. a. Critical point transition analysis for upper and lower paths with cells divided into different numbers of clusters. The critical point index is calculated within each subpopulation from the upper and lower paths respectively and color-coded onto the FLOW-MAP. Red indicates a higher value, and blue represents a lower value. Clusters, shown where labeled, shows the highest critical point index value in the upper or lower path. **b.** Importance score, as defined by node-degree, of each node within each "marker-marker correlation network", networks were calculated from the labeled cell cluster. Colors indicate the node-degree value of each node within networks. Nodes labeled with stars were further-tested with drug perturbation.

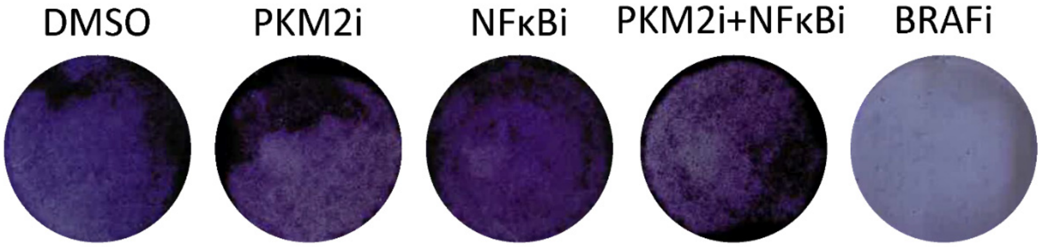

Supplementary Figure 28. Short-term clonogenic assay for 397 cells. M397 was treated with either DMSO control or PKM2i or NFKBi or PKM2i+NFKBi or BRAFi. No significant toxicity to the cells was observed for using PKM2i or NFKBi or combination of both.

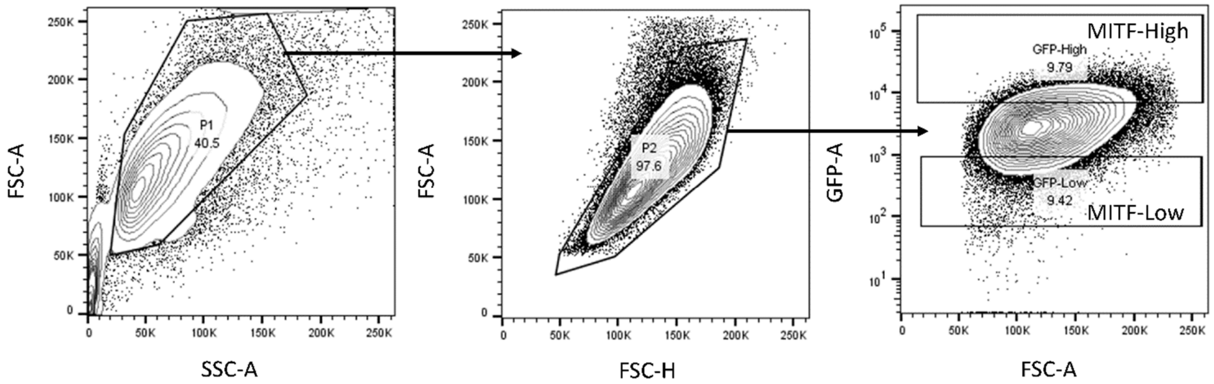

Supplementary Figure 29. Gating strategy for FACS sorting of the MITF-High and MITF-Low subpopulations in untreated M397 cells.

Supplementary Table 1. List of primers used in the study

Primer name	Sequence
PFK-F	GGCAGGAGAATGTGCTGGTCAT
PFK-R	CATAAGCGACAGGCGTCAGTTTC
MITF-F	TGCCCAGGCATGAACACAC
MITF-R	GGGAAAAATACACGCTGTGAG
MART1-F	CACGGCCACTCTTACACCAC
MART1-R	GGAGCATTGGGAACCACAGG
Slug-F	GAGCATTTGCAGACAGGTCA
Slug-R	ACAGCAGCCAGATTCCTCAT
RPL19-F	TCAGGTACAGGCTGTGATACA
RPL19-R	GGGCATAGGTAAGCGGAAGG
