# Supplementary material for: Multi-omic single-cell snapshots reveal multiple independent trajectories to drug tolerance in a melanoma cell line
Source: Nat Commun. 2020 May 11;11:2345. doi: 10.1038/s41467-020-15956-9 (PMC7214418; doi:10.1038/s41467-020-15956-9)
Supplement: Supplementary file 2 — Reporting Summary [file 41467_2020_15956_MOESM2_ESM.pdf]

nature research

Corresponding author(s): James R. Heath and David Baltimore

Last updated by author(s): 03/22/2020

Reporting Summary

Nature Research wishes to improve the reproducibility of the work that we publish. This form provides structure for consistency and transparency in reporting. For further information on Nature Research policies, see Authors & Referees and the Editorial Policy Checklist.

Statistics

For all statistical analyses, confirm that the following items are present in the figure legend, table legend, main text, or Methods section.

n/a	Confirmed	
☒	☐	The exact sample size (*n*) for each experimental group/condition, given as a discrete number and unit of measurement
☐	☒	A statement on whether measurements were taken from distinct samples or whether the same sample was measured repeatedly
☐	☒	The statistical test(s) used AND whether they are one- or two-sided<br>*Only common tests should be described solely by name; describe more complex techniques in the Methods section.*
☒	☐	A description of all covariates tested
☒	☐	A description of any assumptions or corrections, such as tests of normality and adjustment for multiple comparisons
☐	☒	A full description of the statistical parameters including central tendency (e.g. means) or other basic estimates (e.g. regression coefficient) AND variation (e.g. standard deviation) or associated estimates of uncertainty (e.g. confidence intervals)
☐	☒	For null hypothesis testing, the test statistic (e.g. *F*, *t*, *r*) with confidence intervals, effect sizes, degrees of freedom and *P* value noted<br>*Give P values as exact values whenever suitable.*
☒	☐	For Bayesian analysis, information on the choice of priors and Markov chain Monte Carlo settings
☒	☐	For hierarchical and complex designs, identification of the appropriate level for tests and full reporting of outcomes
☒	☐	Estimates of effect sizes (e.g. Cohen's *d*, Pearson's *r*), indicating how they were calculated

Our web collection on statistics for biologists contains articles on many of the points above.

Software and code

Policy information about availability of computer code

Data collection	No software was used to collect data.
Data analysis	GraphPad Prism v6 for statistical analyses. Automated acquisition software (METAMORPH, Molecular Devices) was used to acquire differential interference contrast (DIC) and GFP images which were further analyzed using open-source software ilastik (version 1.3.2) and a custom Python (3.7) code which is available upon request.<br>Custom code for analysis of raw CSV output of SCBC measurements was written in R and has been made available/open-source via GitHub (https://github.com/mesako/Melanoma-Publication). FLOWMAPR R package (version 1.2.0) are available on GitHub (https://github.com/zunderlab/FLOWMAP/). Clustering analysis was produced using the Rclusterpp R package (version 0.2.5) using all default settings. Hmisc R package (version 4.2-0) and igraph R package (version 1.2.4.1) were used for the network analysis.

For manuscripts utilizing custom algorithms or software that are central to the research but not yet described in published literature, software must be made available to editors/reviewers. We strongly encourage code deposition in a community repository (e.g. GitHub). See the Nature Research guidelines for submitting code & software for further information.

Data

Policy information about availability of data

All manuscripts must include a data availability statement. This statement should provide the following information, where applicable:

- Accession codes, unique identifiers, or web links for publicly available datasets
- A list of figures that have associated raw data
- A description of any restrictions on data availability

All the data supporting the findings of this study are available within the article and its supplementary information files and from the corresponding author upon reasonable request. Raw data for underlying Figs. 3c-f, h–i, 5a–e, and Supplementary Figs. 3, 4, 5, 6 and 21A–C are provided in the Source Data file.

Field-specific reporting

Please select the one below that is the best fit for your research. If you are not sure, read the appropriate sections before making your selection.

☒ Life sciences ☐ Behavioural & social sciences ☐ Ecological, evolutionary & environmental sciences

For a reference copy of the document with all sections, see nature.com/documents/nr-reporting-summary-flat.pdf

Life sciences study design

All studies must disclose on these points even when the disclosure is negative.

Sample size	Because this study is not a clinical trial of drugs or a comparative study of groups, there is no sample-size calculation in this study. For in vitro experiments, N=3 was used as the minimum samples for quantification.
Data exclusions	No data exclusions.
Replication	All experiments was repeated at least 3 times and all attempts at replication were successful.
Randomization	Randomization is not relevant to this study since most of the experiments are in vitro cell culture related rather than mouse experiments or clinical trials which requires comparative study of groups.
Blinding	Blinding was not relevant to this study because this study did not involve clinical sample grouping etc.

Reporting for specific materials, systems and methods

We require information from authors about some types of materials, experimental systems and methods used in many studies. Here, indicate whether each material, system or method listed is relevant to your study. If you are not sure if a list item applies to your research, read the appropriate section before selecting a response.

Materials & experimental systems

n/a	Involved in the study	
☐	☒	Antibodies
☐	☒	Eukaryotic cell lines
☒	☐	Palaeontology
☒	☐	Animals and other organisms
☐	☒	Human research participants
☒	☐	Clinical data

Methods

n/a	Involved in the study	
☒	☐	ChIP-seq
☐	☒	Flow cytometry
☒	☐	MRI-based neuroimaging

Antibodies

Antibodies used

Alexa Fluor® 488 Mouse anti-Ki-67 - BD - 561165
Human Human/Mouse Total HIF-1 alpha DuoSet IC ELISA - R&D systems - dyc1935-2
Human Axl APC-conjugated Antibody - R&D systems - FAB154A
NGF R/TNFRSF16 DuoSet ELISA - R&D systems - DY367
Anti-Melan-A antibody, Mouse monoclonal - Sigma Aldrich - M6570
Human Melan-A/MART-1 Antibody - R&D systems - AF8008
Human MITF Antibody - R&D systems - AF5769
Anti-MiTF antibody – abcam - ab80651
Human/Mouse/Rat Phospho-ERK1 (T202/Y204) DuoSet IC ELISA - R&D systems - DYC1825
Human Phospho-Src (Y419) DuoSet IC ELISA - R&D systems - DYC2685
Human/Mouse RelA/NFkB p65 Antibody - R&D systems - MAB5078
Phospho-NF-κB p65 (Ser536) (93H1) Rabbit mAb (Biotinylated) - Cell Signaling – 4025
Human Total Axl DuoSet IC ELISA - R&D - DYC16432
ANTI-SNAI2 antibody produced in mouse - Sigma-Aldrich - SAB1412527
Human/Mouse/Rat Total AMPK alpha 1 DuoSet IC ELISA - R&D systems - DYC3197-2
Human/Mouse/Rat Muscle Phosphofructokinase/PFKM Antibody - R&D systems - AF7687
PFKP (D2E5) Rabbit mAb - Cell Signaling – 12746
Human/Mouse/Rat Acetyl-CoA Carboxylase Antibody - R&D Systems - AF6898
Phospho-Acetyl-CoA Carboxylase (Ser79) Antibody -Cell Signaling- 11818
Phospho-LKB1 (Ser428) Antibody - Cell Signaling - 3482
Human LKB1 Antibody, - R&D Systems - AF8055
Anti-PDK1 Antibody - Abcam - ab110335
PDHK1 Antibody - Cell Signaling - 3820
Human/Mouse/Rat PKM2 Antibody - R&D Systems - AF7244

PKM2 Antibody -Cell Signaling- 4053
Lactate Dehydrogenase B Antibody -Novus Biologicals- NBP1-55415
M01 Lactate Dehydrogenase B Antibody - Novus Biologicals - H00003945
PE anti-human CD271 (NGFR) Antibody - Biolegend - 345106
PE anti-ERK1/2 Phospho (Thr202/Tyr204) Antibody - Biolegend - 369505
Melan-A/MART-1 Antibody (A103) [Alexa Fluor® 647] - Novus Biologicals - NBP2-46603AF647
Multiple lots of the same antibodies were tested and used, results were consistent.

Validation	All antibodies used in this study are commercially available and validated by manufacturers. All validation are provided on the product details page of each commercially available antibody.

Eukaryotic cell lines

Policy information about cell lines

Cell line source(s)	Human melanoma cell line M397 were established from patient's biopsies under University of California, Los Angeles, institutional review board approval #11–003254. HEK-293T Cells purchased from ATCC, Manassas, VA Cat # CRL-3216.
Authentication	Cell lines were periodically authenticated to their early passages using GenePrint® 10 System (Promega, Madison, WI).
Mycoplasma contamination	Cell lines used in this study tested negative for mycoplasma contamination
Commonly misidentified lines (See ICLAC register)	No commonly misidentified lines were used in the study.

Human research participants

Policy information about studies involving human research participants

Population characteristics	Because this study only used one cell line that are previous derived from a melanoma patients (this cell line has also been used by many previous publications) and because this study is not directly related to any clinical trials, the population characteristics are not applicable here.
Recruitment	Because this study only used one cell line that are previous derived from a melanoma patients (this cell line has also been used by many previous publications) and are not directly related to any clinical trials, the recruitment information is not applicable here.
Ethics oversight	Patient-derived melanoma cell line, M397, used in this study was previously generated under UCLA IRB approval # 11–003254.

Note that full information on the approval of the study protocol must also be provided in the manuscript.

Flow Cytometry

Plots

Confirm that:

☒ The axis labels state the marker and fluorochrome used (e.g. CD4-FITC).

☒ The axis scales are clearly visible. Include numbers along axes only for bottom left plot of group (a 'group' is an analysis of identical markers).

☒ All plots are contour plots with outliers or pseudocolor plots.

☒ A numerical value for number of cells or percentage (with statistics) is provided.

Methodology

Sample preparation	Cells were washed and trypsinized from culture plates, following by centrifugation at 500g and 4°C for 5 min to pellet cells. Cell pellets were then resuspended in PBS containing 1%BSA before FACS sorting. The gating strategy is shown in Supplementary Fig 29.
Instrument	FACS: FACS Special Order Research Product, Becton-Dickinson.
Software	Flow cytometry plots were analyzed on FlowJo (Treestar).
Cell population abundance	The top 10% GFP-high and the bottom 10% GFP-low subpopulations were sorted.
Gating strategy	The gating strategy is shown in Supplementary Fig 29.

☒ Tick this box to confirm that a figure exemplifying the gating strategy is provided in the Supplementary Information.
